# Human susceptibility to social influence and its neural correlates are related to perceived vulnerability to extrinsic morbidity risks

**DOI:** 10.1038/s41598-018-31619-8

**Published:** 2018-09-06

**Authors:** Pierre O. Jacquet, Valentin Wyart, Andrea Desantis, Yi-Fang Hsu, Lionel Granjon, Claire Sergent, Florian Waszak

**Affiliations:** 1 Laboratoire de Neurosciences Cognitives (LNC), Département d’Etudes Cognitives, INSERM U960, Ecole Normale Supérieure, PSL Research University, F-75005 Paris, France; 2 Institut Jean Nicod, Département d’Etudes Cognitives, CNRS UMR8129, Ecole Normale Supérieure, PSL Research University, F-75005 Paris, France; 3 Université Paris Descartes, Sorbonne Paris Cité, 75006 Paris, France; 4 Centre National de la Recherche Scientifique, Laboratoire Psychologie de la Perception, UMR 8242, 75006 Paris, France; 5 Département Traitement de l’Information et Systèmes, ONERA, Salon-de-Provence, France; 6 Department of Educational Psychology and Counselling, National Taiwan Normal University, 10610 Taipei, Taiwan

## Abstract

Humans considerably vary in the degree to which they rely on their peers to make decisions. Why? Theoretical models predict that environmental risks shift the cost-benefit trade-off associated with the exploitation of others’ behaviours (public information), yet this idea has received little empirical support. Using computational analyses of behaviour and multivariate decoding of electroencephalographic activity, we test the hypothesis that perceived vulnerability to extrinsic morbidity risks impacts susceptibility to social influence, and investigate whether and how this covariation is reflected in the brain. Data collected from 261 participants tested online revealed that perceived vulnerability to extrinsic morbidity risks is positively associated with susceptibility to follow peers’ opinion in the context of a standard face evaluation task. We found similar results on 17 participants tested in the laboratory, and showed that the sensitivity of EEG signals to public information correlates with the participants’ degree of vulnerability. We further demonstrated that the combination of perceived vulnerability to extrinsic morbidity with decoding sensitivities better predicted social influence scores than each variable taken in isolation. These findings suggest that susceptibility to social influence is partly calibrated by perceived environmental risks, possibly via a tuning of neural mechanisms involved in the processing of public information.

## Introduction

In modern western societies, standing out from the crowd, being ‘special’, is increasingly regarded as a valuable attribute, and people nowadays manage their uniqueness in almost every domain of their life (e.g., work experience, internships, volunteering, travel, fashion and fads, sports and hobbies, social networks, etc.). Yet, independence and individualism is not valued to the same degree in every society, nor at every time in history^1,2^. Pre-industrial Europe, for instance, emphasized the importance of conformity and traditionalism, individuals took pride in following the ‘ancients’, and parents taught their children to be obedient, to revere their elders and to abide by the majority^3^. Obedience and conformity represent important cultural values in some modern societies as well^4–8^. Within societies, individuals also vary in the degree to which they depend on others’ views to make decisions and form opinions^9–13^. Why is that the case? Why in different times and in different places, people display a preference for independence and personal exploration or a preference for exploiting the behaviours, beliefs and attitudes of their peers? Here we put forward the idea that part of this variability can be explained by differences in sensitivity to environmental risks, and that this covariation should also be reflected in the brain.

Relying on public information (or ‘social information’) to make decisions allows an individual to benefit from behaviours, beliefs and attitudes that have already been tried out by her peers. However, this strategy also has an opportunity cost: the individual can miss more optimal, albeit more delayed and uncertain opportunities that might have occurred had she relied on a more independent, personal mode of information gathering^14^. In gregarious species, individuals are thus expected to constantly weigh the costs and benefits of public information use on the one hand and personal mode of information gathering on the other hand^15–18^. However, evolutionary models suggest that these trade-offs could also be calibrated as a function of a number of recurrent environmental pressures that modulate the fitness costs of public information use and personal mode of information gathering, leading to consistent preferences for one or the other strategy^19,20^.

A fundamental environmental pressure which contributed to shape the human phenotype throughout evolutionary history is extrinsic morbidity^21^. Pathogens convey obvious survival and reproductive costs to the contaminated individuals. To counteract these costs, evolution has selected for a number of phenotypic traits which proved helpful in fighting-off diseases (e.g., the immune system). On top of this layer of adaptations, humans also evolved a suite of psychological traits allowing individuals to detect and avoid risks of pathogenic contamination^22^, and by this way prevent a metabolically costly physiological immune response. The degree to which individuals estimate their own vulnerability to pathogens is one of them. People higher in disease concern exhibit a range of conservative behaviours, notably in the social domain^23^. These behaviours can be broadly described as preferences for options which have been experienced enough to provide immediate benefits to the detriment of innovative options that can be acquired from personal exploration, and which provide potentially larger but less certain payoffs. The likely reason is that if the environment conveys risks of pathogenic contamination, then exploring new food resources, objects, locations, or social interactions is likely to impose dramatic fitness costs on the individual. One way to compensate for costs imposed by environmental risks is therefore to acquire skills, knowledge and values on the short run by mimicking peers^24–26^. In sum, perceived vulnerability to extrinsic morbidity risks should shift the cost-benefit trade-off of public information use: the more risky the environment is perceived, the greater the susceptibility to social influence should be.

On top of this, the possibility that perceived vulnerability to extrinsic morbidity risks and susceptibility to social influence might be linked to alterations of brain structure and activity has been entirely neglected so far. The few landmark studies that addressed this issue have shown that susceptibility to social influence positively correlates with grey matter volume in the lateral orbitofrontal cortex^27^ and with an overall increased activity of the dorsal anterior cingulate cortex^28–33^. However, the neural correlates of inter-individual variations in susceptibility to social influence and their adaptive function remain unknown to date^34^. Here we address the hypothesis that a greater vulnerability to extrinsic morbidity risks increases susceptibility to social influence. Given the above mentioned evidence, this positive relationship might be mediated by an increased responsivity of the brain to conflicting feedbacks from peers.

**Figure 1:**
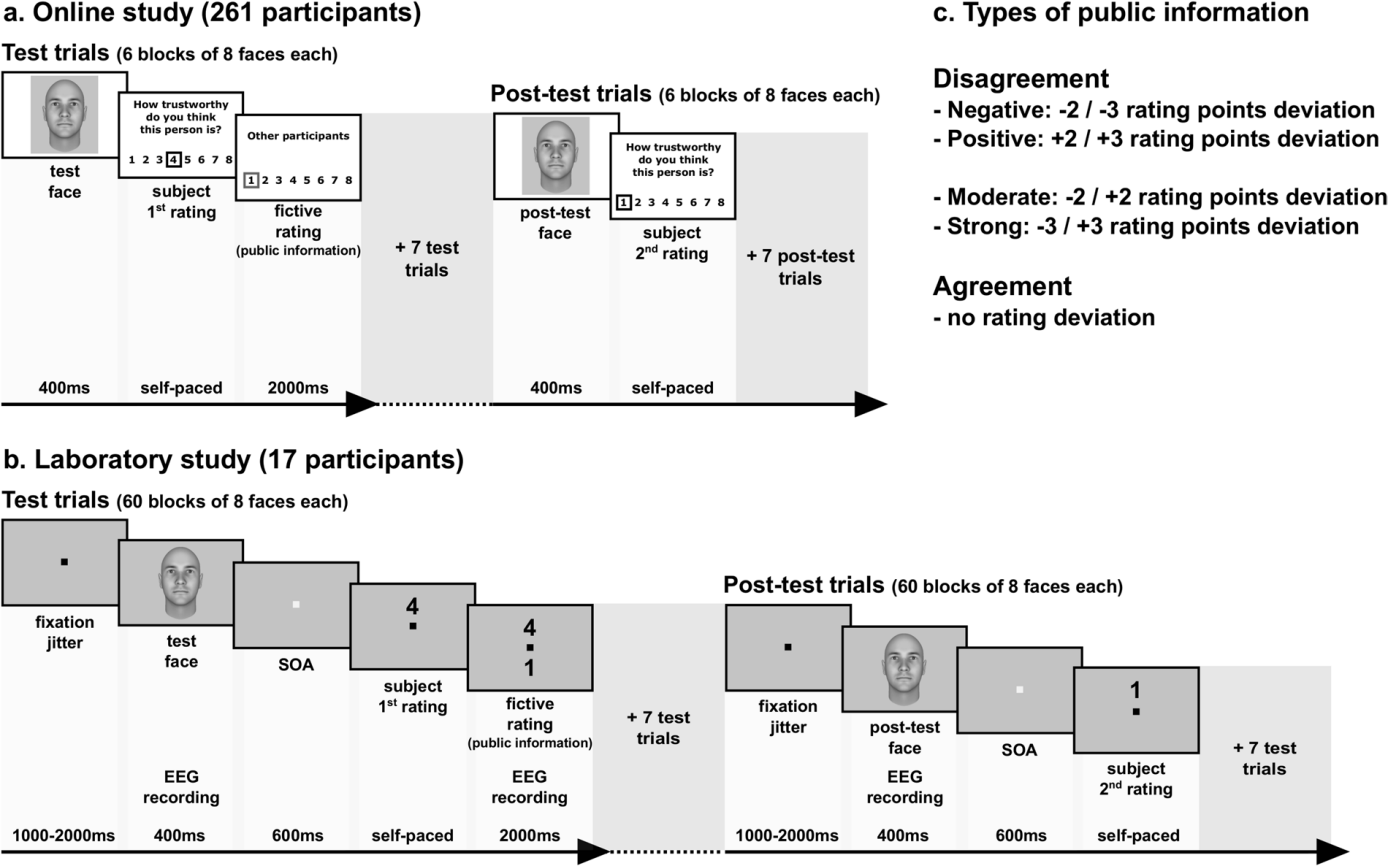
Experimental procedures. (**a**) Online study. In a test trial participants had to rate the computerized face on the trustworthiness dimension using the 8-point scale. Faces were generated following the methods of^80–82^ using the FaceGen Modeller 3.1. The selected value appeared on the scale and is immediately followed by public information (fictive rating). In the example, the fictive rating representing public information is 3 points inferior to the participant’s rating. Six blocks of 8 test trials were interleaved with 6 blocks of 8 post-test trials. In a post-test trial participants were instructed to rate for a second time the trustworthiness of the face they watched in test-trial. In the example, the participant exploited public information to adjust the rating (from 4 in the test trial to 1 in the post-test trial). (**b**) Laboratory study. The structure of the task used in the laboratory was similar as the one used online, but was adapted to the requirements of controlled electroencephalographic recording. Electroencephalographic activity was registered during the presentation of the face in both test and post-test trials (epoch duration: 1200 ms), and during the presentation of public information in test trials (epoch duration: 1200 ms). Sixty blocks of 8 test trials were interleaved with 60 blocks of 8 post-test trials. (**c**) Types of public information.

To test these hypotheses, we adapted a well-validated face evaluation task^29–31,35,36^ that we administered online to 300 participants recruited via a crowdsourcing platform (Amazon Mechanical Turk). Participants were asked to rate unfamiliar faces on the trustworthiness dimension on an 8-point Likert scale before and after watching to the most frequent ratings provided by a fictive group of peers, i.e., the public information (Fig. 1a,b). This rating could positively or negatively deviate from the participants’ rating to a moderate or high extent. Trials in which the public information disagreed with the participants’ ratings can thus be split according to two dimensions: valence (positive or negative deviation) and strength of the disagreement. In another type of trials, the public information matched the participants’ ratings (agreement trials) (Fig. 1c). Scores obtained in the Perceived Infectability and Germ Aversion subscales of the Perceived Vulnerability to Disease questionnaire^37^ were used as standard proxies of perceived vulnerability to extrinsic morbidity risks (the reader will find a detailed description of the questionnaire and a list of its items in the Materials and Methods section). This experimental procedure was also applied in the laboratory with 18 participants while continuously recording their EEG activity (Fig. 1b,c). A canonical computational model of choice^38^ was used in both studies to analyze the weight attributed by participants to public information during post-test trustworthiness ratings (see Supplementary Information). Multivariate decoding was used to analyze the EEG data recorded in the laboratory^39^. A temporal generalization method^40^ was further employed to track the dynamics of the neural ‘coding’ of public information and face processing. Finally, inter-individual variations were tracked by regressing the behavioural and neural correlates of susceptibility to social influence against scores of perceived vulnerability to extrinsic morbidity risks.

## Results

In the following paragraphs, the analyses of data collected online and in the laboratory are presented in a sequential order. The section dedicated to the online study contains a description of the analyses performed on the behavioural data only. The section dedicated to the laboratory study contains a description of the analyses performed on the behavioural data, followed by a description of the results obtained from the multivariate decoding of EEG data.

Analyses of behavioural data acquired online and in the laboratory were performed using Matlab version R2014b and R. Participants’ performance was first analyzed in terms of *mean rating change* (see Supplementary Figure S1 of the Supplementary Information). The mean rating change is the mean difference between the participant’s ratings collected after watching the public information and the participant’s ratings collected before watching the public information. It indicates whether the participant increased (positive value) or decreased (negative value) the trustworthiness ratings after watching the public information. From this mean rating change we calculated a *social influence score* for each participant (see Materials and Methods), and fitted it with a canonical computational model of choice (the full description of the model and the analyses of the fitted parameters are reported in the Supplementary Information, the results are graphically represented in the Supplementary Figures S2, S3, S4 and S5). Positive and negative social influence scores indicate that participants adjusted their trustworthiness ratings towards or away from public information, respectively. Therefore, the greater this score, the greater the participant’s susceptibility to social influence. The effect of public information (i.e., disagreements between the group ratings and the participant’s ratings) and the effect of indicators of perceived vulnerability to extrinsic morbidity risks (Perceived Infectability and Germ Aversion scores) on social influence scores were analyzed with linear mixed models using the *lme* function of the *nlme* R package^41^. All linear mixed models used a maximum likelihood fitting method and had random intercepts. Bayes factors (*BF*_10_) with default Jeffreys-Zellner-Siow (JZS) priors were also calculated using the *lmBF* function of the *BayesFactor* R package to compare the predictive power of the linear mixed models^42^. Comparing models using Bayes factors allowed us to determine whether indicators of perceived vulnerability to extrinsic morbidity risks have an effect on social influence scores and, if yes, whether this effect was greater than the effect of potentially confounding factors like the participants’ age or gender. A Bayes factor superior to 1 indicates greater evidence for the alternative model, while values inferior to 1 indicates greater evidence for the baseline model.

## Online study

### Effect of public disagreement on social influence scores

The effects of public information on social influence scores were analyzed using a linear mixed model taking participants’ ID as a random factor, disagreement valence (negative vs. positive) and strength (moderate vs strong) as within-subject fixed-effect factors. This model served as a baseline for comparison analyses described in the next sections.

**Figure 2:**
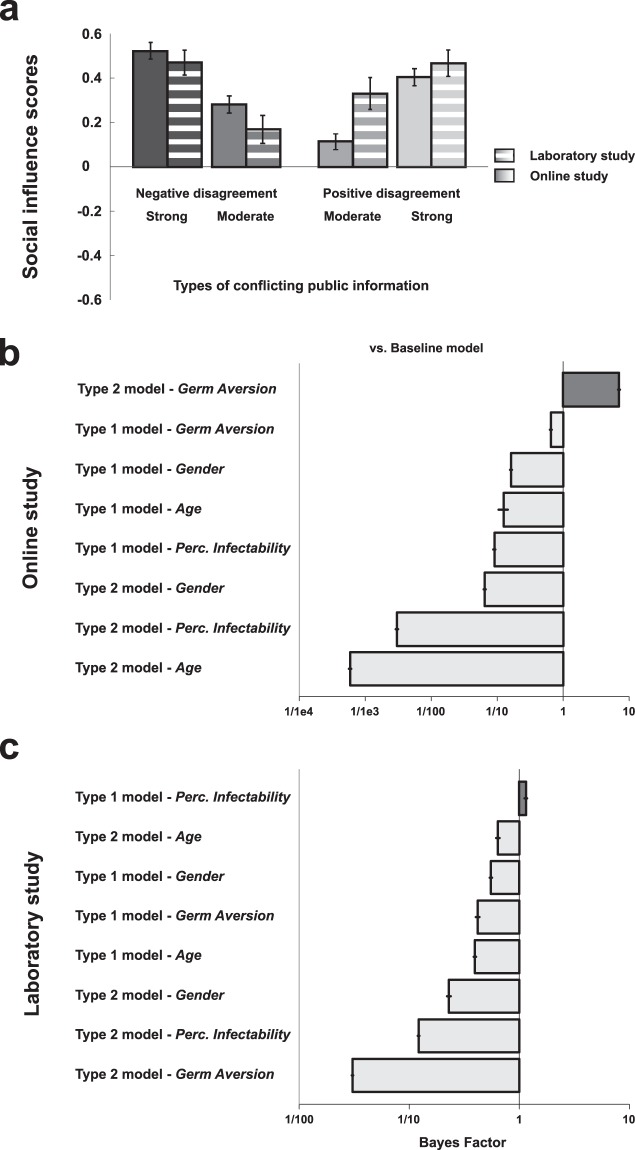
Behavioural results. (**a**) Effects of disagreement types on social influence scores (±SEM) in the online and the laboratory study. Positive and negative social influence scores (y axis) indicate that participants adjusted their ratings towards or away from public information. (**b**) Online study and (**c**) Laboratory study: Bayesian analyses of models with and without indicators of perceived vulnerability to extrinsic morbidity risks (Germ Aversion and Perceived Infectability), age or gender as predictor of social influence score (columns). The baseline model only includes disagreement valence and disagreement strength as within-subject factors; alternative models include indicators of perceived vulnerability to extrinsic morbidity risks, age or gender either as a main effect (type 1) or as a term interacting with disagreement valence and disagreement strength (type 2). A Bayes Factor >1 indicates greater evidence for the alternative model.

Social influence scores of participants tested online were on average greater when exposed to negative disagreements than positive disagreements (*β* = 0.17 ± 0.05, *t*(780) = 3.21, *p* < 0.002), and greater for strong disagreement than moderate disagreements (*β* = 0.29 ± 0.05, *t*(780) = 5.57, *p* < 0.001). The interaction between disagreement valence and disagreement strength was not significant (Fig. 2a).

### Effect of perceived vulnerability to extrinsic morbidity risks on social influence scores

The baseline model above mentioned was then enriched by the inclusion of indicators of perceived vulnerability to extrinsic morbidity risks (Germ Aversion and Perceived Infectability scores). These alternative models were compared with two additional alternative models in which age and gender were respectively added as the between-subject predictor in place of Germ Aversion or Perceived Infectability scores. In each alternative model, the between-subject predictor was either included as a main effect (type 1 models) or as a term interacting with disagreement valence and disagreement strength (type 2 models). Bayes factors (*BF*_10_) were calculated to compare the predictive power of alternative models with the baseline model taken as the reference.

The strongest evidence was found for the type 2 model which included the Germ Aversion score as an indicator of perceived vulnerability to extrinsic morbidity risks (*Germ Aversion* vs. *Baseline*: *BF*_10_ = 7.08 ± 4.98%; *Germ Aversion* vs. *age*: *BF*_10_ = 12078.44 ± 4.91%; *Germ Aversion* vs. *gender*: *BF*_10_ = 109.65 ± 4.96%; Fig. 2b). Germ Aversion score had a positive main effect on social influence scores (*β* = 0.08 ± 0.04, *t*(259) = 2.26, *p* = 0.024), and interacted with disagreement valence and disagreement strength (*β* = −0.18 ± 0.07, *t*(777) = −2.41, *p* = 0.016). This interaction effect was due to the fact that in strong positive disagreement trials exclusively, the Germ Aversion score was negatively related to the social influence score (*β* = −0.08 ± 0.04, *t*(259) = −2.22, *p* = 0.027). In all other types of disagreement, a positive association was found (moderate positive disagreement: *β* = 0.10 ± 0.04, *t*(259) = 2.94, *p* = 0.003; strong negative disagreement: *β* = 0.07 ± 0.04, *t*(259) = 1.94, *p* = 0.053; moderate negative disagreement: *β* = 0.08 ± 0.04, *t*(259) = 2.23, *p* = 0.027). A complementary linear mixed model in which strong positive disagreement trials were excluded, and in which the other types of disagreement trials were coded as a 3-level factor (moderate negative, moderate positive, strong negative), confirmed the main effect of the Germ Aversion score (*β* = 0.09 ± 0.02, *t*(259) = 3.77, *p* < 0.001). Evidence in favour of this complementary model was even greater than the original version (*Germ Aversion* vs. *Baseline*: *BF*_10_ = 52.38 ± 1.43%). However, models which included the Perceived Infectability score as an indicator of perceived vulnerability to extrinsic morbidity risks had a lower predictive power than the baseline model (*BFs* < 1).

Of note is that neither the Germ Aversion score nor the Perceived Infectability score affected the mean rating change in agreement trials (trials in which the public information matched participant’s ratings, see Supplementary Information for details).

## Laboratory Study

### Effect of public disagreement on social influence scores

The effects of public information on social influence scores were analyzed using a linear mixed model taking participants’ ID as a random factor, disagreement valence (negative vs. positive) and strength (moderate vs strong) as within-subject fixed-effect factors. This model served as a baseline for comparison analyses similar to those described in the next sections.

Social influence scores of participants tested in the laboratory were on average greater for strong than moderate disagreements (*β* = 0.30 ± 0.08, *t*(48) = 3.61, *p* < 0.001). No other significant effects were found (Fig. 2a).

### Effect of perceived vulnerability to extrinsic morbidity risks on social influence scores

To match the analytic procedure used in the Online study, the baseline model above mentioned was then enriched by the inclusion of indicators of perceived vulnerability to extrinsic morbidity risks (Germ Aversion and Perceived Infectability). Similarly, these alternative models were compared with two additional alternative models in which age and gender were respectively added as the between-subject predictor in place of Germ Aversion or Perceived Infectability scores. In each alternative model, the between-subject predictor was either included as a main effect (type 1 models) or as a term interacting with disagreement valence and disagreement strength (type 2 models). Bayes factors (*BF*_10_) were calculated to compare the predictive power of alternative models with the baseline model taken as the reference.

The strongest – although small – evidence was found for the Type 1 model taking Perceived Infectability score as indicator of perceived vulnerability to extrinsic morbidity risks (*Perceived Infectability* vs. *Baseline*: *BF*_10_ = 1.15 ± 4.25%; *Perceived Infectability* vs. *age*: *BF*_10_ = 2.91 ± 4.82%; *Perceived Infectability* vs. *gender*: *BF*_10_ = 2.08 ± 4.76%; Fig. 2c). An increase in Perceived Infectability score was associated with an increase in social influence score, and so independently of disagreement valence and disagreement strength (*β* = 0.07 ± 0.03, *t*(15) = 2.30, *p* = 0.036). Note that no models involving the Germ Aversion scores outperformed the baseline model (*BFs* < 1). Germ Aversion scores did not have significant effect on the social influence score, although the relationship between the two variables went in the predicted direction (*β* = 0.03 ± 0.04, *t*(15) = 0.68, *p* = 0.50).

Finally, the two indicators of perceived vulnerability to extrinsic morbidity risks had no effect on the mean rating change obtained in agreement trials (see Supplementary Information for details).

### Decoding public information

**Figure 3:**
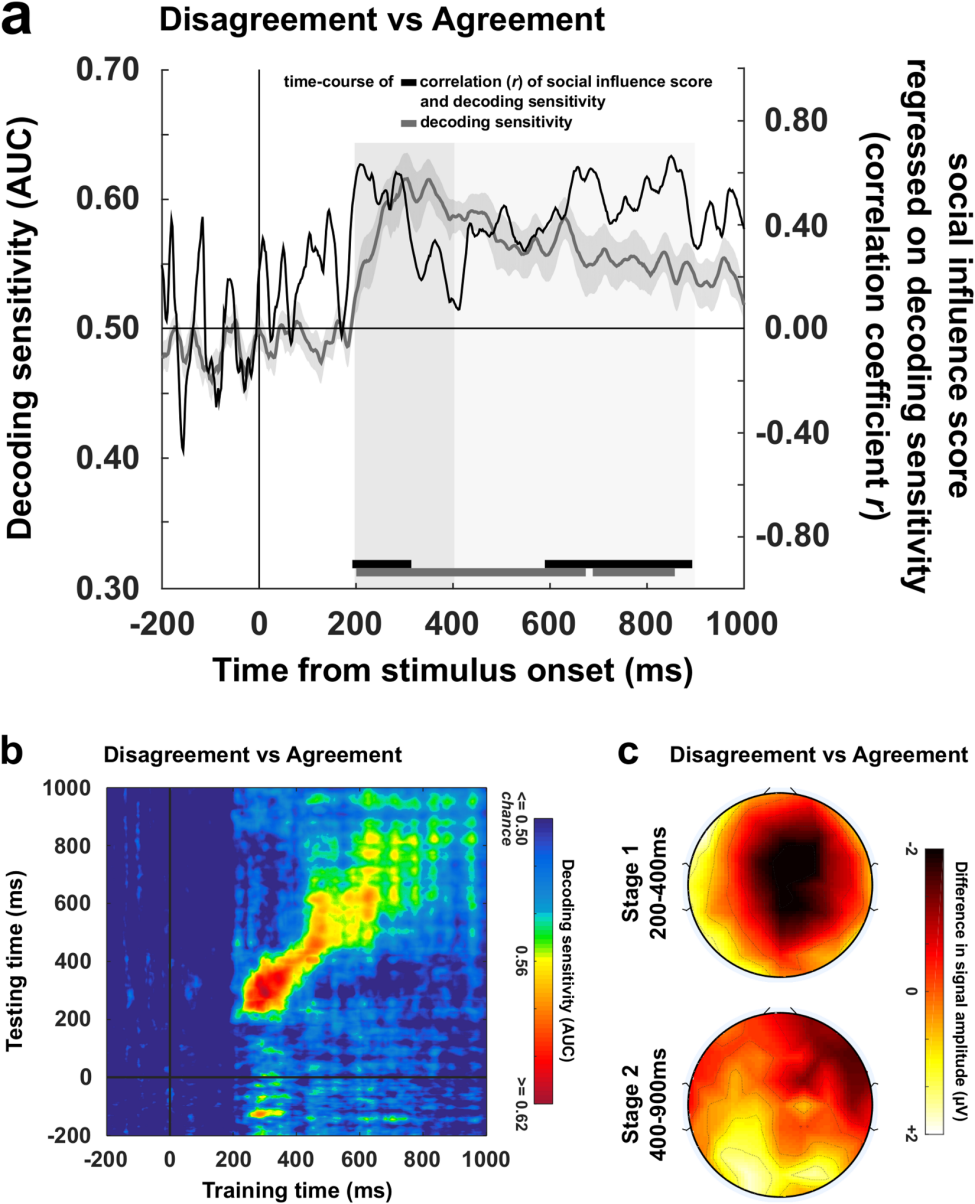
Decoding stages of public information processing and temporal generalization. (**a**) The grey curve represents the sensitivity of the decoder (±SEM) that was trained to classify disagreement trials and agreement trials on the basis of the EEG activity (left *y* axis) recorded during the 1000 ms following the exposure to public information (*x* axis). Disagreement trials are entered in the classification pipeline irrespective of their valence and strength. Clusters of adjacent time-points in which the decoder’s sensitivity significantly differed from chance are represented by the grey markers located up to the *x* axis. The black curve represents the time-course of correlation (coefficient *r*, right *y* axis) between decoding sensitivities of public information processing and social influence scores. Clusters of adjacent time-points in which the correlation coefficient *r* was >0.40 are represented by the black markers located up to the *x* axis. (**b**) Decoders trained at each time point were tested on data from all other time points, revealing the presence of two distinct processing stages (stage 1 = 200–400 ms post-stimulus; stage 2: 400–900 ms post-stimulus). The diagonal (where testing time = training time) gives the curve for canonical decoders performance over time. (**c**) Topographical maps of the differential EEG activity resulting from the contrast between the two classes of stimuli that were entered in each decoder are representative of processing stages 1 and 2.

Several decoders with binary classifications were run to decode the EEG activity (from −200 ms before to 1 s after stimulus onset) evoked by the processing of the various types of public information. Electrodes were used as decoding features (*N* = 64) and decoders were run independently for each time points of an epoch (sampling rate = 500 Hz; *N* = 600). A first decoder was trained to distinguish – or classify – disagreement trials (whatever the valence and strength) from agreement trials. Clusters of adjacent time-points with decoding sensitivities significantly superior to 0.50 (one-tailed *t*-tests, significance threshold = *p* < 0.05) were identified and corrected for multiple comparisons using Monte-Carlo Permutation tests^43^. The significance threshold of decoding clusters was set at = *p < 0.01 (*p is the true proportion of clusters obtained from random data permutations whose *AUC* sums are greater than the *AUC* sum calculated from the real dataset, see Materials and Methods section for details). Figure 3a depicts the time course of the decoder’s sensitivity (area under the curve - *AUC*) averaged across participants. Two successive clusters were identified (cluster 1: *mean AUC* = 0.58, *p < 0.001; cluster 2: *mean AUC* = 0.55, *p < 0.001). These two clusters remarkably covered an important portion of the epoch: the decoding sensitivity differed from chance level 205 ms after the onset of public information, and turned back to chance level 645 ms later.

To determine whether distinct processing stages could be isolated from the dynamics of the decoder performance, we used the ‘temporal generalization’ methods^40^ which consists in testing whether a classifier trained at a certain time point *t* is able to generalize to other time points *t’*. The objective is to reveal whether an extended classification results from a stable pattern of brain activation or if it is made of a sequence of distinct neural codes. If the mental representation is stable, then the classifier should remain efficient even if applied at different latencies. If, instead, the mental representation is successively re-encoded in a series of different brain activations, therefore we should observe a failure of generalization beyond a certain temporal duration (i.e., away from the diagonal were *t* = *t’*). Matrices of temporal generalization revealed that, for the classification of disagreement and agreement trials, decoding was in the close neighbourhood of the diagonal between 200 ms and 400 ms post-stimulus (Fig. 3b). Beyond that point and up to 900 ms on average, classifiers generalized to a wider neighbourhood of latencies (see Supplementary Information), hence confirming the presence of two distinct processing stages of public information. The first stage was characterized by a sharp sensitivity peak around 300 ms post-stimulus (*AUC peak* of 0.62 on average) and was associated with a negative deflection located in the neighbourhood of the fronto-central sites of the scalp surface (Fig. 3c). The second stage was more stationary, covered a wider time-window, and was characterized by (1) a negative differential activity in the vicinity of right frontal electrodes, and by (2) a positive differential activity distributed around occipito-parietal sites (Fig. 3c). While the ERPs associated with the first stage resembled a Feedback-Related Negativity (FRN)^44,45^, the second stage shared characteristics with EEG patterns associated with the encoding and the maintenance of visual information in working memory^46–49^.

What these patterns suggest is that maintaining and manipulating public information in working memory could serve future rating adjustments. If this is the case, then participants exhibiting greater classification scores during the second processing stage should also be more susceptible to social influence. To test this hypothesis we performed complementary correlation analyses in which the participants’ social influence scores were regressed on their decoding sensitivities calculated at each time-point of each processing stage of public information. Clusters of adjacent time-points with correlation coefficient *r* superior to or equal to 0.40 were identified and corrected for multiple comparisons using Monte-Carlo permutation tests^43^. The significance threshold of correlation clusters was set at = *p < 0.05 (*p is the true proportion of clusters obtained from random data permutations whose *r* sums are greater than the *r* sum calculated from the real dataset, see Materials and Methods section for details). We found that social influence score and decoding sensitivity positively correlated during both the first (200 ms–310 ms post-stimulus, *p = 0.015; *mean r* = 0.55) and the second processing stages (595 ms–890 ms post-stimulus, *p = 0.01; *mean r* = 0.52) (Fig. 3a).

Decoding analyses, temporal generalization and correlation analyses implying disagreement valence and disagreement strength are reported in the Supplementary Information (see also Supplementary Figure S6).

### Decoding changes in face processing as a function of public information and type of rating adjustment

Another important challenge of the present study was to investigate whether the brain activity recorded during the evaluation of face trustworthiness could be modulated by the type of public information participants were exposed to and, if yes, whether this modulation further depended on the type of rating that participants made in post-test (i.e., a rating adjusted towards or away from public information). For each type of public information independently, we trained a decoder which classified faces that were judged in test trials (before watching public information) vs faces that were judged in post-test trials (after watching public information). As expected, in the agreement condition the decoder did not performed above chance. Remarkably, classification scores turned significant when the test/post-test contrast concerned faces that were paired with positive disagreements, but not with negative disagreements. This was evidenced by a cluster of time points which covered a wide time-window starting 495 ms and ending 840 ms after the face onset (*mean AUC* = 0.54, *p = 0.001).

**Figure 4:**
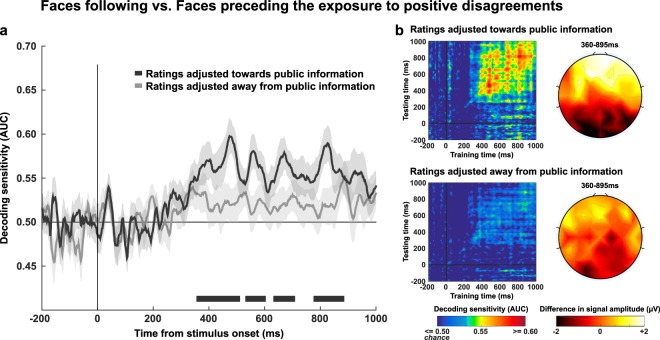
Decoding stages of face processing as a function of subsequent rating adjustment. (**a**) Sensitivity of the decoders that were trained to classify faces presented before vs after a positive disagreement, and which resulted in a rating adjusted towards public information (black curve) or away from public information (light grey curve). Significant clusters are represented by the corresponding markers appearing at the bottom of the graphs. (**b**) The left panels depict the temporal generalization matrices of the decoders performance specific to trials resulting in ratings adjusted towards (upper panel) or away (lower panel) from public information. The diagonal (where testing time = training time) gives the curve for canonical decoders performance over time. The upper and lower right-hand panel represent the topographical maps of the differential EEG activity (faces following-preceding positive disagreements) recorded during trials resulting in ratings adjusted towards or away from public information, respectively. In both contrasts, the differential EEG activity was averaged across time-points composing the entire time-series which included the four clusters of significant decoding sensitivity (from 360 ms to 895 ms post-stimulus).

In order to check whether this successful classification was accounted for by the type of rating adjustment participants made, we performed the same contrast twice: first by targeting trials which resulted in ratings adjusted *towards* public information, second by targeting trials which resulted in ratings adjusted *away* from public information. The decoder significantly performed above chance only for trials which resulted in ratings adjusted towards public information. This pattern was characterized by a sequence of 4 consecutive clusters, starting 360 ms and ending 985 ms after the face onset. The first three clusters were separated by a 25 ms duration on average, and covered a 335 ms duration time-window whose *AUC* values peaked at 0.60 around 500 ms post-stimulus (cluster 1: *mean AUC* = 0.57, *p < 0.001; cluster 2: *mean AUC* = 0.57, *p = 0.014; cluster 3: *mean AUC* = 0.56, *p = 0.007). Finally, a last significant cluster of a 100 ms duration emerged 790 ms after the face onset, with an AUC value peaking at 0.59 (*mean AUC* = 0.57, *p = 0.002) (Fig. 4a). A temporal generalization analysis showed that from 360 ms post-stimulus and up to the end of the epoch, classifiers generalized to a wide neighborhood of latencies. This result provides evidence that the four clusters shared a common brain activation pattern characterized by (1) a sustained negative-going deflection of the surface potential occurring in occipito-parietal sensors and by (2) a greater positivity in fronto-central sites (Fig. 4b). These patterns were not observed with trials which resulted in ratings adjusted away from public information (Fig. 4b).

### Decoding public information as a function of perceived vulnerability to extrinsic morbidity risks

We then tested whether the participants’ scores in the two indicators of perceived vulnerability to extrinsic morbidity risks were associated with the decoder’s performance at the two distinct stages of public information processing described in a previous paragraph. For each processing stage independently, we ran correlation analyses between scores in Perceived Infectability or Germ Aversion alternatively, and the decoding sensitivities computed at each time-point of the epochs. Significant clusters of correlation were identified using the same methods described in a previous section.

**Figure 5:**
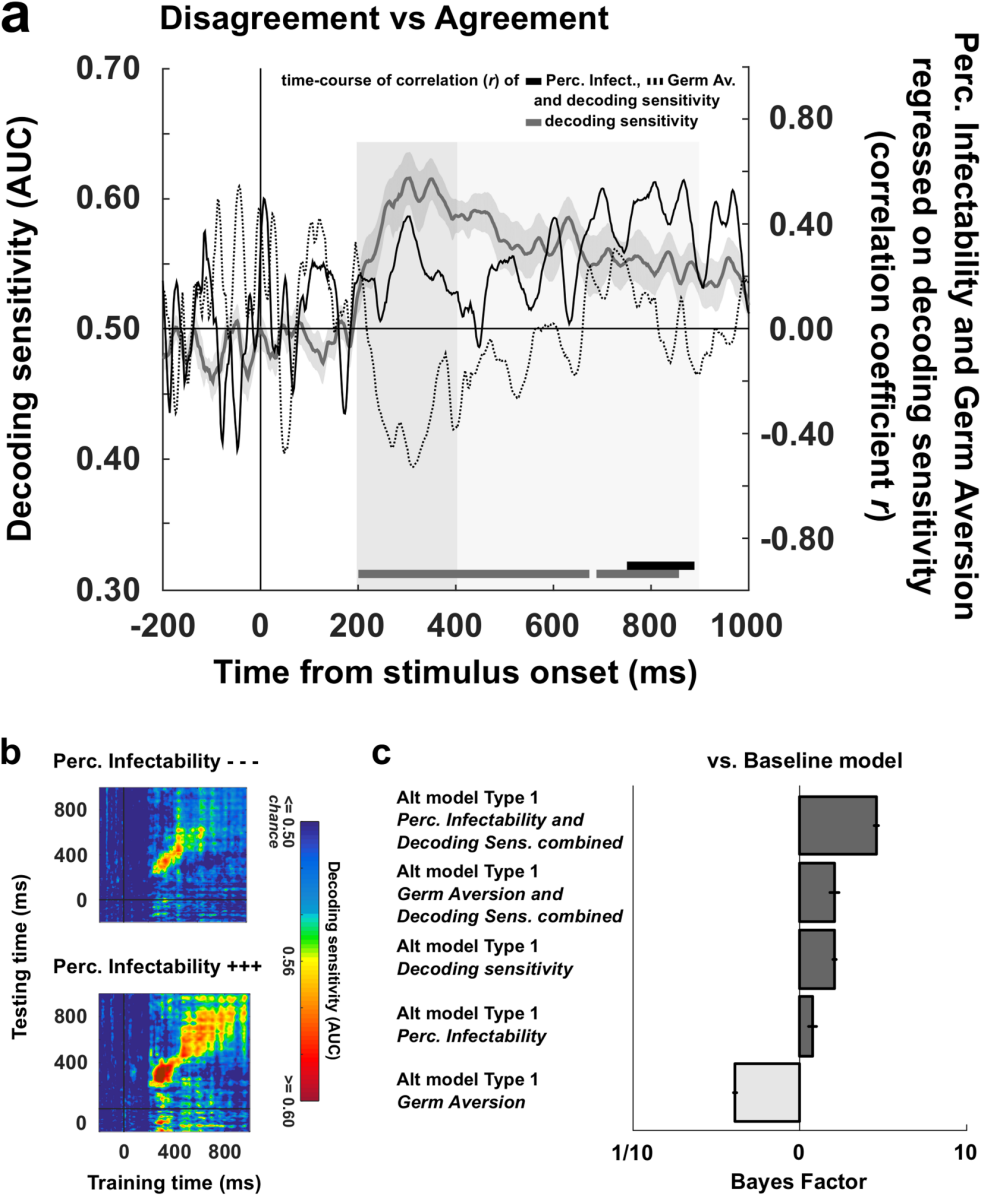
Decoding stages of public information processing as a function of indicators of perceived vulnerability to extrinsic morbidity risks. (**a**) The solid and dotted black curves represent the time-course of correlation (coefficient *r*, right *y* axis) between decoding sensitivities of public information processing (grey curve, left *y* axis) and Perceived Infectability scores on the one hand (solid curve), and Germ Aversion scores on the other hand (dotted curve). Clusters of adjacent time-points in which the correlation coefficient *r* was >0.40 are represented by the corresponding markers located just above the *x* axis. (**b**) Temporal generalization of public information decoding obtained after splitting the participants sample into high and low scorers on the Perceived Infectability subscale (median split). The diagonal (where testing time = training time) gives the curve for canonical decoder performance over time. (**c**) Bayesian analyses of models with and without the index summing Perceived Infectability scores or Germ Aversion scores with decoding sensitivities, or each of these variables taken in isolation as predictor of social influence scores. The baseline model only includes disagreement valence and disagreement strength as within-subject factors; alternative models include the combination indices, decoding sensitivity, Perceived Infectability or Germ Aversion as a main effect (type 1). A Bayes Factor >1 indicates greater evidence for the alternative model.

Cluster analyses revealed that the greater were the Perceived Infectability scores, the greater were the decoding sensitivities (Fig. 5a). This positive relation was shown in a cluster of 128 ms duration emerging 755 ms after the onset of public information (*p = 0.035; mean *r* = 0.48). Temporal generalization analyses further showed that participants who scored high in Perceived Infectability (median split) presented a long-lasting pattern of significant decoding covering both processing stages 1 and 2, with the latter stage characterized by a generalization of the classification pattern to a wide neighbourhood of latencies (Fig. 5b). Although a series of positive correlations emerged between Germ Aversion scores and decoding sensitivities around 700 ms, their amplitude and length were too small to be captured as a significant cluster (Fig. 5a).

Complementary analyses investigating the correlation of scores in Perceived Infectability, Germ Aversion, and decoding sensitivities obtained from the classification of agreement trials and disagreement trials varying in valence and strength are reported in the Supplementary Information.

### Joint contribution of decoding sensitivity and perceived vulnerability to extrinsic morbidity risks to social influence scores

The last analytic step consisted in investigating the joint contribution of decoding sensitivity and indicators of perceived vulnerability to extrinsic morbidity risks to social influence scores. First, we created a combination index in which the AUC score of each participant averaged on both the first and the second stages of public information processing was *z*-transformed and summed with the Perceived Infectability (*z*-)score on the one hand, or with the Germ Aversion (*z*-)score on the other hand. Second, the mean decoding sensitivity and each combination index were successively entered as a main effect into linear mixed models taking social influence scores as the dependent variable, disagreement valence and strength as within-subject factors, and participants’ ID as random factor. Third, Bayes factors (*BF*_10_) were calculated to compare the predictive power of models including either the indicators of perceived vulnerability to extrinsic morbidity risks, either the mean decoding sensitivity, or the combination index. A baseline model including participants’ ID as a random factor, disagreement valence (negative vs. positive) and strength (moderate vs strong) as within-subject fixed-effect factors was taken as the reference for model comparison analyses.

The greater evidence was found for the model including the index combining Perceived Infectability scores and decoding sensitivities as main predictor (*Combination of Perceived Infectability and decoding sensitivity* vs. *Baseline*: *BF*_10_ = 2.88 ± 3.65%; *decoding sensitivity*: *BF*_10_ = 1.61 ± 3.27%; *Perceived Infectability* vs. *Baseline*: *BF*_10_ = 1.19 ± 7.15%) (Fig. 5c). Hence, the greater was the combined index, the greater was the participants’ social influence scores (*β* = 0.06 ± 0.02, *t*(15) = 3.20, *p* = 0.006). Germ Aversion alone decreased the predictive power of the model (*Germ Aversion* vs. *Baseline*: *BF*_10_ = 0.41 ± 7.82%). However, its combination with decoding sensitivity improved the model’s fit (*Combination of Germ Aversion and decoding sensitivity* vs. *Baseline*: *BF*_10_ = 1.62 ± 7.82%). The greater was the score combining Germ Aversion and decoding sensitivity, the greater was the social influence score (*β* = 0.06 ± 0.02, *t*(15) = 2.64, *p* = 0.018).

## Discussion

The present results showed that proxies of perceived vulnerability to extrinsic morbidity risks modulated the extent to which participants were influenced by public information in the context of a standard face evaluation task. This positive association was found in a large cohort of participants tested online as well as in a smaller sample tested in the laboratory. Computational analyses of behavioural data further suggested that the mechanisms leading participants with higher disease concern to be more susceptible to social influence is an increased reliance on public information instead of an increased corruption of their internal representations by noise (see Supplementary Information for a detailed description of the analyses of computational data, and Supplementary Figure S2, S3, S4 and S5 for graphical representations of the results). Furthermore, using multivariate decoding of EEG data we observed distinct, dynamically evolving neural responses that reflected the content of public information as well as its influence on subsequent trustworthiness judgments on the one hand, and on the processing of the characteristics of faces on the other hand. Most importantly, we found that social influence scores and perceived vulnerability to extrinsic morbidity risks both correlated positively with the brain’s responsivity to public information within overlapping time-windows. These findings suggest that, (1) the degree of susceptibility to social influence might be calibrated by the individual’s perception of extrinsic morbidity risks, and that (2) these positive association might be mediated by an increased responsivity of the brain to conflicting feedbacks from peers. This view is further supported by the fact that, in the laboratory study, the combination of scores in perceived vulnerability to extrinsic morbidity risks and decoding sensitivities better predicted the inter-individual variation in social influence score than each variable taken in isolation.

In line with previous research using a similar peer-pressure procedure^29–31,35,36^, we showed that, on average, participants tested online as well as those tested in the laboratory aligned their ratings on public information when the feedbacks it provided conflicted with the participants’ social preferences. This alignment was more pronounced for trials paired with a strong than a moderate disagreement. These results were adequately replicated by a canonical computational model of choice. In addition, classification of EEG data collected in the laboratory remarkably matched behavioural data. Our decoders revealed extended differences between EEG patterns coding for public agreement and EEG patterns coding for public disagreement. Notably, decoders had better performance for strong disagreement trials than moderate disagreement trials, and performed equally well for positive and negative disagreement trials. Temporal generalization analyses^40^ showed that, overall, disagreement trials elicited two distinct processing stages. The first stage was characterized by a negative activity within the fronto-central sites of the scalp surface between 200 ms and 400 ms after stimulus onset. This is coherent with a previous study showing that the exposure to a disagreement with a group of peers elicited a negative prediction error coded by a feedback-related negativity (FRN)^30^. However, we also showed that EEG patterns coding for disagreement and agreement trials could differ at later processing stages (400 ms–900 ms), with an increased positive activity in the left occipito-parietal electrodes coupled with an increased negative activity in the right prefrontal sites. Similar differences in EEG activity have been shown to predict differences in visual working-memory demands^46–49^. Interestingly, we found that both the social influence score calculated from real data and the social influence parameter fitted by our computational model correlated with the decoding sensitivity during this late processing stage. It suggests that the alignment of a rating on public information observed in a post-test trial is a decision which is made right after the processing of public disagreement, possibly via an active encoding of the relevant information into working memory.

Another important challenge of the current study was to investigate whether the brain activity recorded during the evaluation of faces could be modulated by the various types of public information they were paired with and, if yes, whether this modulation depended on the type of ratings participants made in post-test trials (i.e., adjusted or not to public information). Results revealed that the decoders did not performed above chance for faces paired with agreement trials. Decoding sensitivities turned however significant when the test/post-test contrast concerned faces paired with positive disagreement trials. This test/post-test modulation of face processing had a strategic outcome: it occurred only in trials ending in ratings adjusted towards public information. Temporal generalization analyses showed that the EEG activation pattern of post-test faces processing was homogeneous and long-lasting. Compared to pre-test faces indeed, this pattern was characterized by a pronounced ‘early posterior negativity’ (EPN) over occipito-parietal sensors and a ‘late positive potential’ (LPP) of greater amplitude in fronto-central sites, i.e., two components indicative of an amplified or prioritized processing of emotional attributes of visual stimuli^50–54^. These results is in striking contrast with the fact that the social influence score correlated with the decoding of public information in the negative disagreement condition only, not in the positive disagreement condition (see Supplementary Information). How to account for this dissociation? We propose that it might result from an error-management problem^55–58^. In both the online and the laboratory studies indeed, the participants’ initial ratings were slightly moved towards the untrustworthiness side of the scale (online study: *M* = 4.27, *t*(260) = 92.40, *p* < 0.001; laboratory study: *M* = 4.15, *t*(16) = 30.79, *p* < 0.001). Error-management theory predicts that negative decision biases of this kind result from an asymmetry of costs between false-positive and false-negative errors, with false-positive errors conveying greater fitness costs than false-negative errors. Judging an unfamiliar person as trustworthy on first sight increases the likelihood of having a mutually advantageous interaction with that person but simultaneously increases the risk of being harmed, cheated, exploited, or exposed to another type of threat (e.g., pathogenic contamination). Conversely, judging an unfamiliar person as untrustworthy on first sight conveys a reversed trade-off. In line with this view, our participants prioritized the least costly option as a first-line choice, and this preference was further reinforced by the exposure to negative disagreements. This might explain why later decoding stages of public information positively predicted social influence scores in negative disagreement trials only. Errors associated with negative ratings being on average less costly, participants took no additional risk in aligning with negative disagreements, and therefore could make this decision as soon as public information was processed. Conversely, aligning one’s own ratings to match positive disagreements conveys higher costs in case this decision proved flawed. To make such a decision however, one might take advantage of other opportunities for gathering new pieces of evidence about the trustworthiness of the person. In our experiment, such additional opportunities are offered to the participants by the post-test trials. This view might help explain (1) why social influence scores and the decoding patterns of positive disagreement trials did not correlate, and (2) why the decoding patterns of faces were affected only in trials which involved a positive disagreement and which ended in a rating adjusted towards public information.

On top of these findings, the most important result in our view is that the participants’ social influence scores on the one hand, and the neurophysiological dynamics observed during the processing of public information on the other hand, positively correlated with how much vulnerable to extrinsic morbidity risks they felt. Both the online and the laboratory studies showed that participants who were particularly responsive to extrinsic morbidity risks were on average more susceptible than others to align their ratings on public information. In the laboratory study, this behavioural pattern was correlated with a greater responsivity of the brain to public disagreements. Interestingly, we also found that the combination of scores in perceived vulnerability to extrinsic morbidity risks and sensitivities of the decoder to disagreement trials better predicted social influence scores than each variable taken in isolation. These results suggest that perceived vulnerability to extrinsic morbidity risks increases susceptibility to social influence, possibly via an upregulation of the neural signal triggered by the exposure to conflicting feedbacks from peers.

Overall, these results are in line with recent works performed at the country level^8^ as well as the individual level^59,60^. These works bring evidence that variations in some correlates of social influence – i.e., conformist attitudes and behaviour, importance of obedience, collectivistic attitudes and beliefs – reflect adaptive responses to variations in extrinsic risks (objective as well as subjective) of pathogenic contamination. Nevertheless, some differences should be noticed between these works and our own contribution. More specifically, in the study that we administered online, there was one type of public information in which the positive association between social influence scores and perceived vulnerability to extrinsic morbidity risks was not found, i.e., the strong positive disagreement condition. Quite the contrary in fact: in this specific condition the two variables were negatively linked. One might see in this result an additional exemplification of the error-management problem described in the previous paragraph. Following error-management theory, one might follow other people’s opinion regarding the trustworthiness of an unfamiliar person at the condition that the associated cost is manageable. Judging an unfamiliar person as untrustworthy on first sight is a decision which, if flawed, is less costly than judging an unfamiliar person as trustworthy on first sight. So if the cost of error is too high, then the optimal policy is to stop following the peers’ opinion. According to this approach, a possible interpretation of our result is that the estimation of the asymmetry of errors’ cost might be biased by the degree of vulnerability to pathogens perceived by the participants. In other words, the more the participants feel vulnerable to pathogen threats, the riskier it is for her to follow the most positive evaluation made by the group about someone that they judged more negatively on first sight. This interpretation is fueled by a recent work showing that participants with higher degrees of perceived vulnerability to extrinsic morbidity risks exhibited greater behavioural avoidance tendencies when viewing photographs of unknown people faces^61^.

These observations address the question of the specificity of the effect of perceived vulnerability to morbidity risks on susceptibility to social influence. Does this effect specifically apply to decisions in the social domain? A few recent studies offer tentative answers. Notably, Wu and Chang^60^ showed that participants with higher disease concern (measured with the PVD scale or manipulated thanks to a priming techniques) were more likely than others to conform to the opinions, attitudes, or behaviours of the majority. But most importantly, the stimuli and the psychometric scales they used to assess the participants’ conformist attitudes and behaviours were typically non-social (e.g., esthetic judgements about abstract paintings). Similarly, Murray and Schaller^59^ found that priming participants with morbidity cues elicited a greater alignment with the majority opinion in a non-social task (i.e., student participants had to indicate whether they agreed or disagreed with a potential scenario in which their university might change the numerical scale on which course grades are reported on student transcripts). These results provide pieces of evidence in support of the idea that the impact of perceived vulnerability to morbidity risks on susceptibility to social influence is likely to generalize to non-social decision-making domains^59,60^.

Our two studies and their results also include some limitations. The most important of which is that the indicator of perceived vulnerability to morbidity risks which correlates with social influence scores differed according to the study (i.e., the Germ Aversion score in the online study; the Perceived Infectability score in the laboratory study). Germ Aversion measures an individual’s discomfort in situations implying a heightened likelihood of pathogen transmission. Perceived Infectability measures the individual’s subjective susceptibility to be infected by pathogens. The literature reports that both indicators must covary in the same way with a number of other psychological traits (i.e., health anxiety, illness attitudes, disgust sensitivity, agreeableness, extraversion, neuroticism and openness, socio-sexual orientation, belief in a dangerous world)^37^, and both undoubtedly captured part of our vulnerability to morbidity risks. Then how to account for the difference between our two sets of results? First of all, it is important to remind that the few studies which investigated a similar topic also provided mixed results, so our case is not unique^59,60^. This being said, a possibility is that people experiencing a significant feeling of disgust in situations implying a risk of pathogen transmission (what measures the Germ Aversion indicator) are more widespread in the general population than people who have high concern about the efficiency of their physiological immune system (what measures the Perceived Infectability indicator). We might have been lucky enough to test such a sample in the laboratory study but not in the online study (Perceived Infectability scores were on average slightly higher in the laboratory sample than in the online sample: 3.4 vs. 3.1). Another possibility is that data acquired online are noisier overall. The absence of association between Perceived Infectability scores and social influence scores could be due to the fact that performance in the task could have been corrupted by noise. An additional and non-exclusive possibility is that the very poor inter-item reliability of the Germ Aversion subscale extracted from the laboratory sample could indicate the presence of an important noise component in these data, hence reducing our chance of observing an association with social influence scores. In any cases, these observations highlight the need to test the replicability of our findings in the future, putting extra efforts into better controlling for potential sources of random noise (i.e., refining the experiment to overcome the limitations of online procedures; reducing as much as possible the possibility of a sampling bias together with dealing with constraints of EEG experiments).

As a final point of the discussion, we sought to raise the issue of the causes of the inter-individual variation in vulnerability to morbidity risks and, by extension, in susceptibility to social influence. Part of this variation is likely to be attributable to genetic factors^62–66^. With regard to personality traits for instance (e.g., the five factors model of personality), heritability quantified by common methods of behavioural genetics is usually estimated between 30% and 50%^67^. It means that 50% to 70% of the variance in personality traits and subtraits like perceived vulnerability to extrinsic morbidity risks is not captured by direct genetic influences. Two main alternatives can then be considered. First, the unexplained population variance simply reflects noise. Second, there is a certain amount of signal in it. This would imply that part of the unexplained variance could reflect a set of optimal phenotypes developing at the scale of an individual’s lifespan in response to different environmental states. In other words, experiencing an environment with elevated risks of extrinsic morbidity might trigger a set of psychological mechanisms adapted to the detection of such risks on the one hand (e.g., increased perceived vulnerability), and adapted to the production of risk-avoidance behaviours on the other hand (e.g., increased susceptibility to social influence).

An open question is whether an increased susceptibility to social influence is specifically unfold in condition of high extrinsic morbidity risks. Evolutionary models state that increased susceptibility to social influence should be observed once the environment is too risky to be explored individually. And after all, environmental risks recurrently met by our ancestors throughout the evolutionary history are not confined to pathogens. Therefore, it is reasonable to take into account the possibility that susceptibility to social influence could vary depending on the harshness of the environment in general rather than on the extrinsic morbidity rate alone. Here ‘harshness’ can be understood as the sum of environmental variables (as well as their fluctuations in space and time) which impose costs on the survival and the reproductive success of individuals (e.g., ambient pathogens, food or economic resources, social capital, parental investment). Interestingly, environmental harshness has been shown to alter individuals’ behaviour in significant ways^21,24,68,69^. An increasing number of evidence suggests that harsh and unpredictable environments trigger an adaptive search for immediate instead of long-term benefits in various domains such as health^70^, reproduction^71^, parenting^72^, economic decision-making^73^ or cooperation^74^. In addition, recent data suggest that cumulative adversity experienced up to adulthood – a variable that conceptually overlaps with exposure to environmental harshness – leads to significant changes in the structure of the medial prefrontal and anterior cingulate cortices as well as in the insula^75–79^. This findings are of primary importance. Indeed, these regions are likely to be the sources of the neurophysiological dynamics of public information processing that we recorded in the present study, providing an additional support for the plausibility of a ‘phenotypic plasticity hypothesis’ of susceptibility to social influence.

In conclusion, our findings are in line with recent proposals^9–13^ which challenge the classical view of public information use as a flexible mechanism that individuals optimally exploit according to prevailing circumstances. To the best of our knowledge however, the present work is the first to reveal functional variations in responsivity to environmental risks, brain activity involved in the encoding of public information, and susceptibility to social influence.

## Materials and Methods

In the following paragraphs the reader will find a section dedicated to the description of the materials and methods used in the online study, followed by a section dedicated to the description of the materials and methods used in the laboratory study.

Note that in both studies, the experiment was presented to the participants as a social perception task whose aim was to understand how people built social judgements from unknown faces. Therefore, the experimenters never overtly informed the subjects that the real purpose of the experiment was to investigate the psychological and/or the neurophysiological correlates of susceptibility to social influence. The protocols used online and in the laboratory were approved by the local Ethical Committee (Conseil d’évaluation éthique pour les recherches en santé – CERES n°201659 and n°201313), and were in accordance with the Declaration of Helsinki (World Medical Association, 2008).

## Online Study

### Participants

Three-hundred US participants were initially recruited online via Amazon Mechanical Turk, and 298 participants finally completed the study. Mechanical Turk is an internet crowdsourcing platform through which users can be paid to complete online tasks, including surveys and experiments. The goal of the experiment here was to compare ratings of trustworthiness in response to computerized human faces before (test rating trials) and after (post-test rating trials) participants have been confronted with the modal rating provided by a fictive group of peers (public information). All participants (159 females; mean age: 34 years ± 11) reported being naïve to the purpose of the experiment, gave their written informed consent and received payment for their participation in accordance with the standards of Mechanical Turk.

### Stimuli and procedure

Stimuli consisted of emotionally neutral faces (N = 48; size = 477 × 400 pixels) obtained from the FaceGen Modeller 3.1 (Singular Inversions, 2007) open database developed by Todorov and colleagues^80–82^. Each face has a unique identity, although all of them were male, bald, Caucasian, front facing and with direct gaze. Contrasts were therefore approximately identical across faces. The experimental procedure was adapted from Klucharev and collaborators^29^, and was composed of two types of trials distributed into interleaved blocks (N = 6) of 8 trials each. In a block of test trial (Fig. 1a), participants watched a series of 8 faces each presented for 400 ms, a duration that has been shown to minimize participants’ ability to encode the identity of the faces but that provides enough time to consistently estimate social traits^83–85^. After the presentation of each face, participants were required to rate it on the trustworthiness dimension using an 8-point scale (by clicking on the computer mouse), from 1 = very untrustworthy to 8 = very trustworthy. Once the value was selected, the group rating appeared on the screen for a duration of 2000 ms (Fig. 1a). This ‘public information’ was described to participants as other participants’ modal rating. A block of test trials was followed by a block of post-test trials, in which participants were asked to rate for a second time the same 8 faces previously seen (but presented in a different order) (Fig. 1a). The completion of the online task lasted approximately 9 min.

### Public information

Unbeknownst to the participants, the rating presented as the modal rating provided by a group of other participants was in fact fictive and generated on-line by means of a simple algorithm. We stayed as elusive as possible about the identities of the individuals who composed the fictive group of reference as well as on their number. Providing detailed information about the individuals’ identities could bias the participants’ responses as a function of the affiliation or non-affiliation they feel^86^. In addition, beyond a certain group size (typically 5–10 individuals) the strength of the influence exerted by a group of reference on the individuals’ decisions made in experimental settings has been shown to be relatively stationary^87^. In line with these findings, we only informed participants that in some trials they will be presented with the modal rating provided by ‘other members of the MTurk community’. A number of previous studies using a roughly similar procedure has shown that participants were convinced that the so-called public information was provided by real individuals^29–31,35,36^. Public information included agreement trials (N = 12) in which the fictive rating matched the participant’s initial rating, and disagreement trials equally split between four possible outcomes (N = 24). In disagreement trials the fictive rating was either higher than the participant’s initial rating (positive disagreement) or lower (negative disagreement); and the deviation was either moderate (+2/−2 points deviation) or strong (+3/−3 points deviation). In sum, disagreement trials varied in terms of disagreement valence (positive vs. negative) and disagreement strength (moderate vs. strong), following a 2 × 2 design. We also introduced additional trials without public feedbacks (a question mark appeared on the screen). These no feedback trials (N = 12) were used as a positive control test to ensure that the participants’ rating changes were indeed motivated by the need of matching public information (see below).

### Assessing susceptibility to social influence

In a first step, we examined the extent to which participants changed their trustworthiness ratings by looking at the mean difference between test and post-test ratings (see Supplementary Information for a full description of the results). Mean rating change was computed in no feedback trials, in agreement trials, and in each type of disagreement trials (moderate positive disagreement, strong positive disagreement, moderate negative disagreement, strong negative disagreement). Positive and negative mean rating change, respectively, indicate that participants increase and decrease their trustworthiness ratings in post-test trials. Social influence was defined as a case where participants adjusted their post-test rating in the direction of public information (e.g., a positive mean rating change in positive disagreement trials and a negative mean rating change in negative disagreement trials). In order to obtain a social influence score that enables the statistical comparison of the effects of the four types of disagreement on participants’ performance, we simply reversed the sign of the mean rating change obtained in negative disagreement trials. A positive score obtained in either positive or negative disagreement trials now uniformly indicates that participants adjusted their ratings towards public information. Conversely, a negative score indicates that participants adjusted their ratings away from public information. Thus, the greater the social influence score, the greater the participant’s susceptibility to social influence.

### Quality check and positive control test

All phases of the online study (the informed consent, the pilot task and questionnaires) were coded using Qualtrics (Qualtrics, Provo, UT, ©2016) and presented in a web browser. We subjected each participant data set to a series of quality control checks ensuring that (1) the participant had declared being 18 years old or more, (2) had entered the correct verification code (generated by Qualtrics at the end of the procedure), (3) did the task only once (by comparing the participants’ IP addresses and the GPS coordinates provided by Qualtrics), (4) had valid data in each experimental condition, and (5) did not put into question the fact that the group rating came from real peers. A number of 287 participants fulfilled these criteria. Of the 287 participants, eleven individuals were outliers on social influence score obtained in disagreement trials (using the ±2 SD from the mean criterion). These eleven participants were discarded from subsequent analyses. A quick look at the mean rating change collected in the no feedback trials revealed that several of the 276 remaining participants showed a biased rating change. In the no feedback trials however, a participant should present an unbiased rating change, i.e., a change whose mean is near-zero and whose distribution is centered on zero. If the rating change in the no feedback trials is positively or negatively biased, then it indicates that the participant changes her rating criterion irrespective of whether public information is present or absent. The rating change in the no feedback trials averaged on the 276 participants clearly differed from zero (*M* = 0.24, *SD* = 0.47, *t* = 8.39, *p* < 0.001). Fifteen participants were outliers on this measure (using the ±2 SD from the mean criterion) and were therefore excluded from the final statistical analyses, performed on a final sample of 261 participants.

### Assessing perceived vulnerability to extrinsic morbidity risks

For each participant, we used the scores obtained at the subscales composing the Perceived Vulnerability to Disease (PVD) questionnaire^37^ as proxies for perceived vulnerability to extrinsic morbidity risks. This questionnaire lists a number of behaviours and attitudes that one might adopt to cope with external morbidity threats, here represented by risks of infection by pathogenic agents (see Table 1 for a full list of items). The first subscale – Perceived Infectability – contains items assessing beliefs about vulnerability to future health problems that may arise as the consequence of the exposure to infectious disease transmission situations. The second subscale – Germ aversion – lists a number of aversive responses to situations connoting the potential transmission of infectious diseases. Higher scores in the Germ Aversion and Perceived Infectability subscales are both indicators of an enhanced vulnerability to extrinsic morbidity risks, as perceived by the participant. An inter-item reliability analysis performed on the item scores collected on the 261 participants that were finally included in the online study showed that both subscales presented a satisfactory internal consistency (Perceived Infectability: Cronbach’s alpha = 0.90; Germ Aversion: Cronbach’s alpha = 0.77). These scores were z-transformed and each of them was used as an independent predictor of social influence scores.

**Table 1:** List of the 15 items composing the perceived vulnerability to disease (PVD) scale and its two dimensions (Perceived infectability, Germ aversion).

	Perceived Infectability	**Germ Aversion**
**1. It really bothers me when people sneeze without covering their mouths**.		☑
2. If an illness is ‘going around’, I will get it.	☑	
**3. I am comfortable sharing a water bottle with a friend. (reverse-scored)**		☑
**4. I do not like to write with a pencil someone else has obviously chewed on**.		☑
5. My past experiences make me believe I am not likely to get sick even when my friends are sick. (reverse-scored)	☑	
6. I have a history of susceptibility to infectious disease.	☑	
**7. I prefer to wash my hands pretty soon after shaking someone’s hand**.		☑
8. In general, I am very susceptible to colds, flu and other infectious diseases.	☑	
**9. I dislike wearing used clothes because you do not know what the last person who wore it was like**.		☑
10. I am more likely than the people around me to catch an infectious disease.	☑	
**11. My hands do not feel dirty after touching money. (reverse-scored)**		☑
12. I am unlikely to catch a cold, flu or other illness, even if it is ‘going around’. (reverse-scored)	☑	
**13. It does not make me anxious to be around sick people. (reverse-scored)**		☑
14. My immune system protects me from most illnesses that other people get. (reverse-scored)	☑	
**15. I avoid using public telephones because of the risk that I may catch something from the previous user**.		☑

## Laboratory study

### Participants

Eighteen healthy adults (9 females, median age: 23 years, range: 19–35 years) took part in the laboratory study. All participants were naïve to the purpose of the experiment, reported normal or corrected-to-normal visual acuity, and none of them reported any neurological, psychiatric, or other medical problems. They gave their written informed consent and received payment for their participation. One participant discovered the real purpose of the study at the very beginning of the task and explicitly reported that public information was fake. This participant was automatically excluded from the behavioural and the EEG analyses.

### Stimuli and procedure

The stimuli and procedure used in the laboratory were similar to those used in the online study. Emotionally neutral faces were specifically generated for the purpose of this experiment using FaceGen Modeller 3.1 (Singular Inversions, 2007) according to the methods of Todorov and colleagues^80–82^. Each face has a unique identity, although all of them were male, bald, Caucasian, front facing and with direct gaze. Contrasts were therefore approximately identical across faces. The number of faces (N = 480) and pairs of interleaved blocks (N = 60) were increased in order to match the standards of electroencephalographic recording studies (Fig. 1b). As a consequence, the completion of the task lasted approximately 90 min (participants could make a pause after each block of post-test trials). Participants sat in a comfortable chair in a dimly lit sound attenuated room. The experiment was conducted using Matlab (MathWorks) with the psychophysics toolbox^88^, with face stimuli (size = 700 × 700 pixels) presented on a 21 in. monitor (resolution = 1980 × 1080) at a distance of approximately 60 cm (subtending 18° of visual angle). Participants were first submitted to a training session consisting in one block of 8 test trials followed by one block of 8 post-test trials. Then the task began. In order to minimize the contamination of electroencephalographic (EEG) activity by uncontrolled visual artifacts, we paid a particular attention to homogenize the shapes, the colors and the spatial configuration of elements characterizing the different screens composing a trial (Fig. 1b). We used a grey background color for all screens, and presented the participants’ ratings and the fictive ratings representing public information in the form of single numbers which respectively appeared up to or down a fixation square located at the center of the screen (Fig. 1b). The 8 values of the trustworthiness scale were distributed on the horizontal numerical scale of the keyboard. Scale values ranging from 1 to 4 and from 5 to 8 (1 = not trustworthy at all; 8 = very trustworthy) were mapped onto the four digits of the left hand and the four digits of the right hand (thumbs excluded), respectively (participants were trained to master the mapping prior to the experiment). Finally, we introduced a fixation jitter (1000 ms–2000 ms) at the beginning of each trial (test and post-test) and an SOA (stimulus onset asynchrony) comprised between 0 and 600 ms between the offset of the face presentation and the onset of the participant’s rating screen. Electroencephalographic activity was registered during the presentation of the face in both test and post-test trials (epoch duration: 1000 ms), and during the presentation of public information in test trials (epoch duration: 1000 ms).

### Public information

The manipulation and factorization of public information in the laboratory matched those used in the online study. In the laboratory study subjects were told that our team already piloted some of the face stimuli (in some trials no feedbacks were associated with the faces, see below) with a group of 20 students, and that they will be presented with these ratings during the task (no matter whether they intend to exploit them or not). A post-experiment verbal debrief revealed that all participants were unaware of the real purpose of the experiment, and found public information plausible. None of them overtly reported being influenced by it. Just like in the online study, public information included agreement trials (N = 120) and disagreement trials equally split between the same four possible outcomes (N = 240). Therefore, disagreement trials again varied in terms of disagreement valence (positive vs. negative) and disagreement strength (moderate vs. strong) in a 2 × 2 design. Trials without public feedbacks were also introduced (N = 120) and used as a positive control test, but were not included in the analyses of behavioural and EEG data.

### Assessing susceptibility to social influence

Mean rating change and social influence scores were defined and calculated by using the same methods described above. Positive and negative mean rating change, respectively, indicate that participants increased and decreased their trustworthiness ratings in post-test trials. A positive social influence score obtained in either positive or negative disagreement trials indicates that participants adjusted their ratings towards public information. Conversely, a negative social influence score indicates that participants adjusted their ratings away from public information. Thus, the greater the social influence score, the greater the participant’s susceptibility to social influence.

### Quality check and positive control test of laboratory data

In the laboratory study, the rating change following the no feedback trials and averaged across participants did not differ from zero (*M* = 0.04, *SD* = 0.17, *t* = 0.95, *p* = 0.36). Therefore no additional subject was excluded.

### Assessing perceived vulnerability to extrinsic morbidity risks

As in the online study, we used the participants’ scores obtained at the subscales composing the Perceived Vulnerability to Disease (PVD) questionnaire^37^ as proxies for perceived vulnerability to extrinsic morbidity risks. A inter-item reliability analysis performed on scores collected on the 17 participants showed that only the Perceived Infectability subscale had a satisfactory internal consistency (Cronbach’s alpha = 0.92). Internal consistency of the Germ aversion subscale was on the contrary unacceptable (Cronbach’s alpha = 0.52). We therefore maximized the scale reliability by sequentially removing the items which had a detrimental impact on the coherence of the construct^89^. After the removal of 4 items (items 4, 9, 11, and 15 of the PVD questionnaire), the Cronbach’s alpha reached a satisfactory value of 0.77. The Germ Aversion score reported in the analyses of the laboratory data therefore represents the mean of the scores obtained at items 1, 3, 7, and 13 of the PVD questionnaire. The Perceived Infectability and the ‘maximized’ Germ Aversion scores were z-transformed and each of them was used as an independent predictor of social influence scores.

### EEG recordings and preprocessing

EEG data collected on the 17 participants who complemented the task in the laboratory were recorded with 64 electrodes (actiCAP, Brain Products GmbH, Germany). The EEG signal was digitized at 500 Hz. EEG analysis was conducted using EEGLAB^90^ and custom built Matlab scripts. The data were band-pass filtered online at 0.01–100 Hz, and low-pass filtered offline at 20 Hz. The data were recomputed to average reference off line. Epochs corresponding to faces and public information presentation were generated from −200 to 1000 ms relative to stimulus onset, with a 200 ms pre-stimulus baseline correction. Ocular artifact correction was conducted in EEGLAB in Matlab using independent component analysis^90^. Following removal of eye blinks and eye movements, artefact rejection was conducted in a semiautomatic manner (in EEGLAB) by rejecting epochs with activity above 100 mV or below −100 mV (*mean % of discarded trials* = 5.3, *SD* = 3.76). Any channels that contributed to the rejection of more than 25% of the total number of epochs were replaced by an interpolated weighted average from surrounding electrodes.

### Multivariate decoding of EEG data

Electrodes were used as decoding features (N = 64) and the decoders were run independently for each time points of an epoch (N = 600). This allowed us to reconstruct for each trial the entire time course of classification sensitivity and study more precisely the dynamics of cognitive states involved in the processing of the various contents of public information and of the faces it was paired with (see Supplementary Information for a full description of the decoding analyses of face processing). We used a stratified *k*-folding method in order to balance the proportion of each stimulus class within the data folds and hence increase the generalization capacity of the classification to unknown data. Data were split into 10 folds, with each fold randomly composed of a testing data set (10% of the data) and a training data set (90% of the data). The testing data sets were compared to the training data sets and trials belonging to the latter were classified according to their probability of belonging to one of the two stimulus classes composing the test data sets. Classification scores across trials and time points were estimated for each subject with a receiver operating characteristic (ROC) curve analysis applied to the obtained classification probabilities and were summarized by the area under the curve (*AUC*) values^39^. The ROC curve presents the true-positive rate (the proportion of trials belonging to stimulus class A and classified as A, i.e., hit rate) as a function of the false-positive rate (i.e., the proportion of trials belonging to stimulus class B and classified as A, i.e., false alarm rate). Importantly, *AUC* analysis provides an unbiased measure of decoding accuracy, robust to imbalanced problems and independent of the statistical distribution of the classes. The *AUC* value of classification was computed for the obtained decoding time series separately for each time point and each participant, and was then averaged across them. A cluster-based analysis with Monte-Carlo Permutations^43^ performed on the time series of individual decoding probabilities was used to determine the moments at which the decoders performed above chance (time points whose *AUC* values significantly differed from 0.5). Using this method we were able to identify clusters of time points in which the two learned stimulus classes significantly differ while correcting for multiple comparisons. For each time point, *p values of the difference between the two decoded classes were first computed by means of a one-tailed *t*-test. Clusters were then identified by taking all dyads of time points adjacent in time with *p* < 0.05. The final significance of the cluster was determined by computing the sum of *AUC* values of the entire cluster and comparing it with the results of the Monte Carlo permutations (1000 permutations)^39^. Clusters were considered significant at corrected *p < 0.01 if the probability of obtaining a cluster with a greater sum of *AUC* values across the permutations was inferior to or equal to 1%. Thus, the reported *p*-value is an exact value (here denoted *p) which corresponds to this probability.

### Correlation of perceived vulnerability to extrinsic morbidity risks with decoding sensitivities

In order to investigate whether the participants’ perceived vulnerability to extrinsic morbidity risks was associated with the neurophysiological dynamics covering the processing of public information, we ran correlation analyses between the decoder’s sensitivity computed at each time-point of the time-series classifying disagreement and agreement trials and the scores obtained in the Perceived Infectability subscale on the one hand, and the Germ Aversion subscale on the other hand. To correct for multiple comparisons, the distribution across participants of each indicator’s score was randomly permuted 10000 times and the correlation was performed on each permutation and at each point of the time series. Clusters of time-points in which the decoder performance significantly correlated with an indicator’s score were determined by using the methods of Maris & Oostenveld^43^ already described in several sections of this manuscript. Clusters were first identified by taking all dyads of time points adjacent in time with a correlation where the coefficient *r* was superior to or equal to 0.40. We then computed the sum of *r* coefficients of the entire cluster and compared it with the results of the permutations. Clusters were considered significant at *p < 0.05 if the probability of obtaining a cluster with a greater sum of *r* coefficients across the permutations was inferior to 5% (the reported *p-value is an exact value which corresponds to this probability). We expected the Perceived Infectability scores and the Germ Aversion scores to positively correlate with the decoding sensitivities.

### Data Accessibility

Data included in this study, as well as the Matlab and R scripts used for their processing, are available through the Open Science Framework at: https://osf.io/s75tq/.

## Electronic supplementary material

Supplementary Information
