# Supplementary material for: Human susceptibility to social influence and its neural correlates are related to perceived vulnerability to extrinsic morbidity risks
Source: Sci Rep. 2018 Sep 6;8:13347. doi: 10.1038/s41598-018-31619-8 (PMC6127093; doi:10.1038/s41598-018-31619-8)
Supplement: Supplementary file 1 — Supplementary Information [file 41598_2018_31619_MOESM1_ESM.pdf]

Human susceptibility to social influence and its neural correlates are related to perceived vulnerability to extrinsic morbidity risks

Pierre O. Jacquet[1,2,3,4], Valentin Wyart[1], Andrea Desantis[3,4,5], Yi-Fang Hsu[3,4,6], Lionel Granjon[3,4], Claire Sergent[3,4], Florian Waszak[3,4].

[1] Laboratoire de Neurosciences Cognitives (LNC), Département d'Etudes Cognitives, INSERM U960, Ecole Normale Supérieure, PSL Research University, F-75005 Paris, France.

[2] Institut Jean Nicod, Département d'Etudes Cognitives, CNRS UMR8129, Ecole Normale Supérieure, PSL Research University, F-75005 Paris, France.

[3] Université Paris Descartes, Sorbonne Paris Cité, 75006 Paris, France.

[4] Centre National de la Recherche Scientifique, Laboratoire Psychologie de la Perception, UMR 8242, 75006 Paris, France.

[5] Département Traitement de l'Information et Systèmes, ONERA, Salon-de-Provence, France

[6] Department of Educational Psychology and Counselling, National Taiwan Normal University, 10610 Taipei, Taiwan.

Supplementary Information

Complementary results

Behavioural data

Effect of public information on mean rating change. As a starting point, we tested whether evaluations of face trustworthiness produced by the 261 participants who satisfied the inclusion criterion of the online study and by the 17 participants who satisfied the inclusion criterion of the laboratory study, changed after the exposure to the evaluations provided by the fictive group of peers (public information). We thus examined how much participants' changed their trustworthiness ratings by looking at the mean difference between their test and post-test ratings. For participants of both the online and the laboratory studies, we computed this rating change in agreement trials as well as in trials where the group rating was either higher than the participant's initial rating (positive disagreement) or lower (negative disagreement); and the deviation was either moderate (+2/-2 points deviation) or strong (+3/-3 points deviation).

Online study. A first look at the online data indicates that the mean rating change differed from zero in each type of trials (moderate negative disagreement: M = -0.28 ± 0.61; strong negative disagreement: M = -0.52 ± 0.60; moderate positive disagreement: M = 0.11 ± 0.58; strong positive disagreement: M = 0.40 ± 0.61; agreement: M = -0.09 ± 0.41; all ts > 3.15, all ps < .001). Importantly, the mean rating change obtained in each type of disagreement trials differed from the mean rating change obtained in agreement trials (all ts > 4.71, all ps < .001). Thus, participants on average biased their rating more when exposed to public disagreement than when exposed to public agreement (Supplementary Figure S1).

Laboratory study. Participants who performed the experiment in the laboratory presented the same pattern than those recruited online (Supplementary Figure S1). Mean rating change differed from zero in trials featuring negative disagreements (moderate: M = -0.17 ± 0.26, t = 2.72, p = .015; strong: M = -0.47 ± 0.23, t = 8.48, p < .001), positive disagreements (moderate: M = 0.32 ± 0.30, t = 4.61, p < .001; strong: M = 0.47 ± 0.25, t = 7.81, p < .001), and in trials

displaying public agreement ($M = 0.12 \pm 0.22$, $t = 2.35$, $p = .032$). The mean rating change obtained in all types of disagreement but the negative disagreement of moderate strength differed from the mean rating change obtained in agreement trials (all ts > 2.35, all ps < .05).

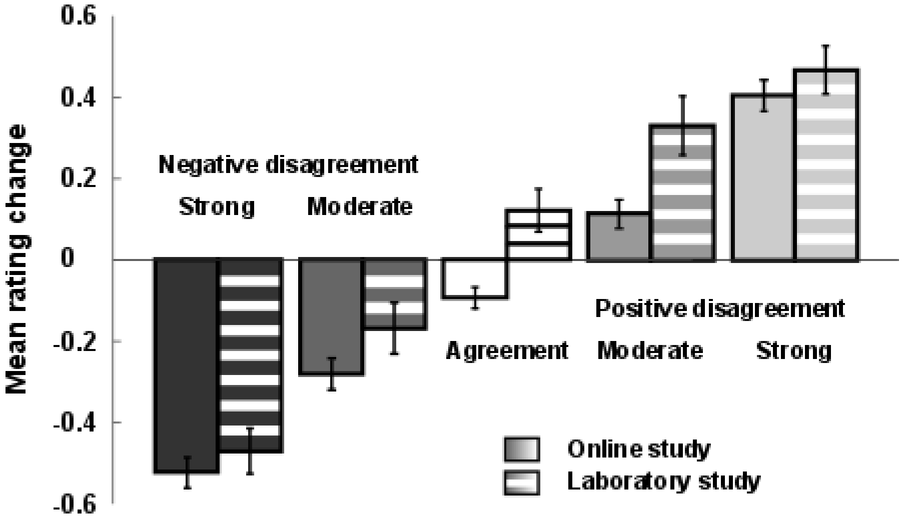

Supplementary Figure S1. Effects of disagreement types on mean rating change (±SEM) in the online and the laboratory study. Positive and negative mean rating changes (y axis) indicate that participants increase or decrease their trustworthiness ratings along the task.

Effect of indicators of perceived vulnerability to extrinsic morbidity risks on mean rating change calculated in agreement trials. A Bayesian analyses showed no evidence that indicators of perceived vulnerability to extrinsic morbidity risks used in the online study and in the laboratory study as well affected the mean rating change calculated in agreement trials. ***Online study.*** Models including either the Germ Aversion score or the Perceived Infectability score had a lower predictive power than a null model including the intercept only (*Germ Aversion* vs. *null*: $BF_{10} = 0.34 \pm 0.79\%$; *Perceived Infectability* vs. *null*: $BF_{10} = 0.32 \pm 0.83\%$). ***Laboratory study.*** Similarly, the null model which included the intercept only outperformed the models involving the Germ Aversion and the Perceived Infectability scores as predictors (*Germ Aversion* vs. *null*: $BF_{10} = 0.63 \pm 0.47\%$; *Perceived Infectability* vs. *null*: $BF_{10} = 0.76 \pm 0.37\%$).

Computational analyses of behavioural data

In the context of the present task, individuals who felt vulnerable to extrinsic morbidity risks may rely on public information not because they overvalue it, but because they may for example exhibit impaired working memory capacities making their representations noisier. To disentangle these two possibilities, we fitted participants' behaviour using a canonical model of choice.

Computational model description and fitting. The fitted computational model hypothesizes that the decision of adjusting a rating after the integration of public information are formed on the basis of a comparison between the faces presented in post-test trials and public information presented in test trials. The model consisted of two free parameters, fitted to each participant's behaviour: 1) a social influence parameter δ corresponding to the adjustment of an initial rating in post-test trials (superior to zero for adjustments in line with public information, equal to or inferior to zero for adjustments independent of public information), measured as the signed fraction of disagreement between the initial rating and the subsequent rating representing public information, and 2) an internal noise magnitude parameter σ corresponding to the standard deviation of the post-test rating. The mean rating in post-test trials μ thus corresponds to a linear combination between the initial rating x_{ini} and the group rating x_{group} following:

$$\mu = x_{\text{ini}} \cdot (1 - \delta) + x_{\text{group}} \cdot \delta$$

The probability of choosing the discrete rating x in post-test trials can be computed using the following equation:

$$p(x) = \Phi\left(x + \frac{1}{2}, \mu, \sigma\right) - \Phi\left(x - \frac{1}{2}, \mu, \sigma\right)$$

where $\Phi(\,.\,)$ is the cumulative normal density function.

We obtained maximum-likelihood estimates of the two parameters δ and σ separately for each participant's behaviour using gradient descent of the negative model likelihood using the 'interior-point' algorithm of the *fmincon* routine implemented in Matlab (Mathworks, Natick, MA). We derived model predictions in terms of social influence scores for all measures made directly from participants' behaviour, as means to test the adequacy of the model.

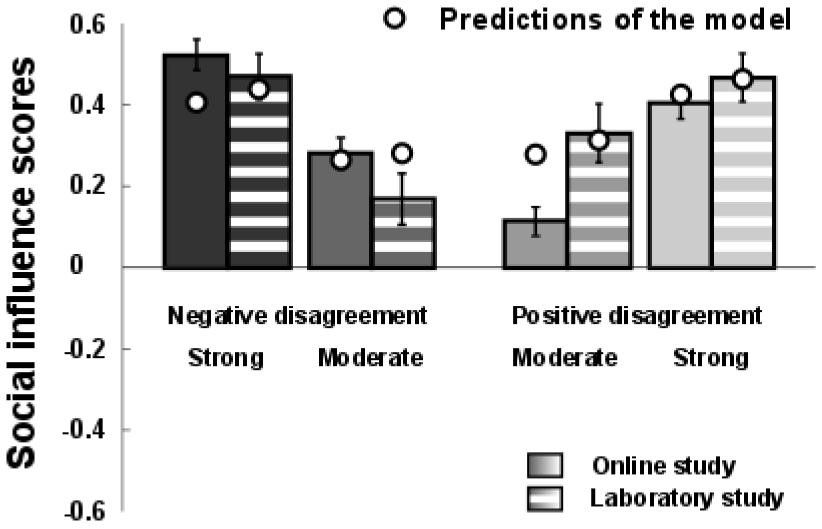

Supplementary Figure S2. Effects of disagreement types on social influence scores (±SEM) in the online and the laboratory study. Positive and negative social influence scores (y axis) indicate that participants adjusted their ratings towards or away from public information. The discs represent the predictions of the computational model for each type of disagreement (see Material and Methods for details).

Testing the adequacy of the computational model. All the effects revealed by the analyses of the participants' mean rating changes calculated from data collected online as well as in the laboratory were replicated by the model's predictions (Supplementary Figure S2).

Online study. First, mean rating change predicted in each type of disagreement trials were greater than the mean rating change predicted in agreement trials (all ts > 15.94, all ps = < .001). Second, the model predicted a social influence score that positively differed from zero for negative disagreements of both moderate (M = .28 ± .11, $t(16)$ = 10.30, p < .001) and bigger strength (M = .44 ± .17, $t(16)$ = 10.47, p < .001) (Supplementary Figure S2). The model

also predicted a greater social influence score for strong negative disagreement trials compared to moderate negative disagreement trials ($t(520) = 5.39$, $p < .001$). Conversely, the model predicted a social influence score that positively differed from zero for positive disagreements of both moderate ($M = .29 \pm .24$, $t(260) = 18.86$, $p < .001$) and bigger strength ($M = .43 \pm .35$, $t(260) = 19.56$, $p < .001$), the latter condition leading to a greater score than the former condition ($t(520) = 5.58$, $p < .001$) (Supplementary Figure S2).

As we just saw the model well predicted the sign of the social influence score in each disagreement type. However the size of the scores observed in the participants differed from its modelled counterpart in two types of disagreement: strong negative disagreement (observed: $M = .52 \pm .60$ vs. modelled: $M = .41 \pm .35$; $t(520) = 2.68$, $p = .008$), and moderate positive disagreement (observed: $M = .11 \pm .58$ vs. modelled: $M = .28 \pm .24$; $t(520) = -4.23$, $p < .001$).

The adequacy of the computational model, comprising only two free parameters, was further evidenced by the amount of inter-individual variance of the observed social influence scores averaged across all disagreement types that was captured by the model-predicted scores ($R^2 = .92$, $p < .001$) (Supplementary figure S3.a). This was also observed in each disagreement type: negative disagreement of moderate ($R^2 = .19$, $p < .001$) and big strength ($R^2 = .40$, $p < .001$) as well as positive disagreement of moderate ($R^2 = .16$, $p < .001$) and big strength ($R^2 = .24$, $p < .001$) (supplementary figure S4).

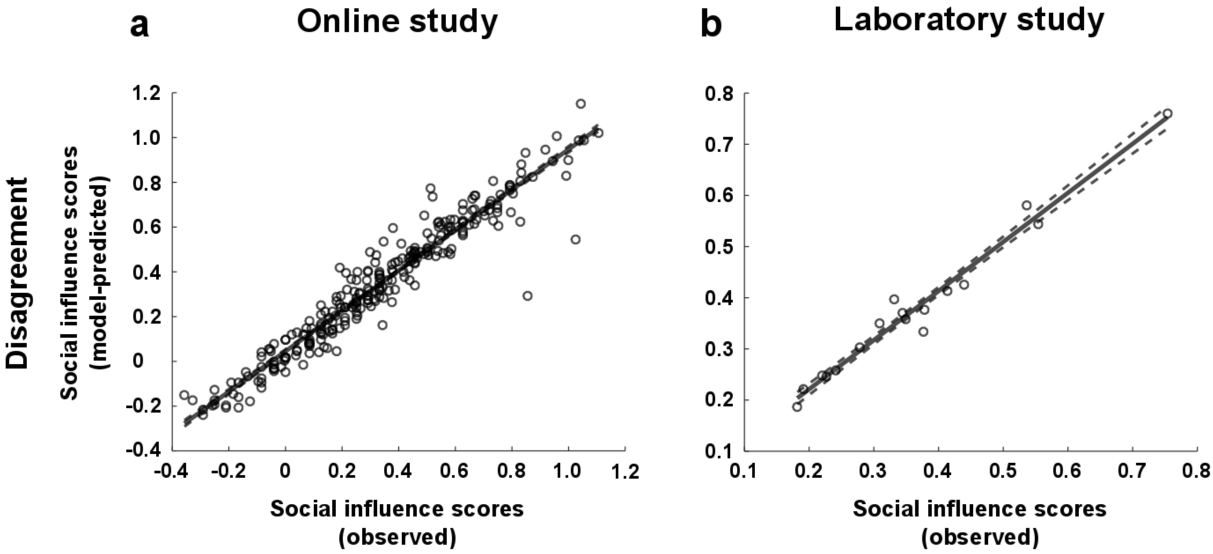

Supplementary Figure S3. Observed social influence scores average across all types of disagreement regressed on predictions of the computational model. a. Online study. **b.** Laboratory study.

Laboratory study. Mean rating change predicted in each type of disagreement trials were greater than the mean rating change predicted in agreement trials (all *t*s > 7.52, all *p*s = < .001). In addition, the model predicted a social influence score that positively differed from zero for negative disagreements of both moderate (M = .28 ± .11, $t(16) = 10.30$, $p < .001$) and bigger strength (M = .44 ± .17, $t(16) = 10.47$, $p < .001$) (Supplementary Figure S2). The model also predicted a greater social influence score for strong negative disagreement trials compared to moderate negative disagreement trials ($t(32) = 3.15$, $p = .004$). The model also predicted a social influence score that positively differed from zero for positive disagreements of both moderate (M = .32 ± .13, $t(16) = 10.02$, $p < .001$) and bigger strength (M = .46 ± .18, $t(16) = 10.78$, $p < .001$), the latter condition leading to a greater score than the former condition ($t(32) = 2.78$, $p = .009$) (Supplementary Figure S2).

The computational model well predicted the sign of the social influence score observed in each disagreement type but, remarkably, also predicted its magnitude in each condition. The adequacy of the computational model was further evidenced by the amount of inter-individual variance of the observed social influence scores averaged across all disagreement

types that was captured by the model-predicted scores (R^2 = .97, p < .001) (Supplementary figure S3.b). This was also the case when the different disagreement types were analyzed separately: negative disagreement of moderate (R^2 = .18, p = .05) and big strength (R^2 = .24, p = .03) as well as positive disagreement of moderate (laboratory study: R^2 = .39, p = .004) and big strength (laboratory study: R^2 = .23, p = .03) (supplementary figure S4).

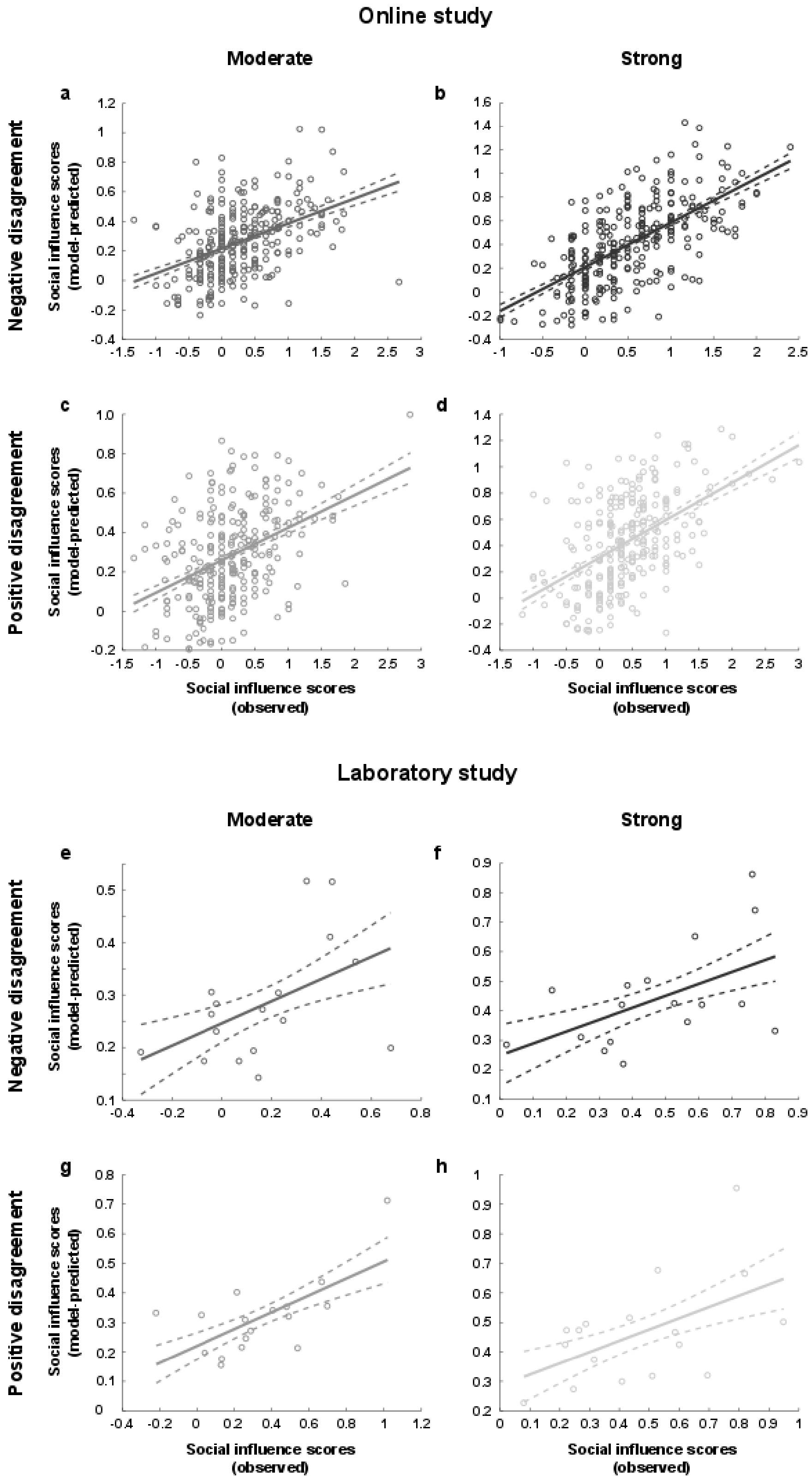

Supplementary Figure S4. Observed social influence scores regressed on predictions of the computational model for each type of disagreement. Online study: a. moderate negative disagreement. **b.** strong negative disagreement. **c.** moderate positive disagreement. **d.** strong positive disagreement**. Laboratory study: e.** moderate negative disagreement. **f.** strong negative disagreement. **g.** moderate positive disagreement. **h.** strong positive disagreement**.**

Effect of indicators of perceived vulnerability to extrinsic morbidity risks on the social influence parameter δ and the noise parameter σ

We then conducted an analysis comparing a baseline model with type 1 and type 2 models including indicators of perceived vulnerability to extrinsic morbidity risks (Germ Aversion and Perceived Infectability), age or gender as predictor. Each models took successively δ or σ as the dependent variable.

Online study. When the social influence parameter δ was taken as the dependent variable, the type 2 model including Germ Aversion as the indicator of perceived vulnerability to extrinsic morbidity risks was the only model that outperformed the baseline model. (*Germ Aversion* vs. *Baseline*: BF_{10} = 3.92 ± 7.81%) (Supplementary Figure S5.a). This model also outperformed type 2 models in which age or gender was entered as the interaction term (*Germ Aversion* vs. *age*: BF_{10} = 4495.49 ± 4.62%; *Germ Aversion* vs. *gender*: BF_{10} = 89.11 ± 4.19%). Model parameters indicated that the effect of the Germ Aversion score had a main effect on the social influence parameter δ (β = 0.04 ± 0.02, $t(259)$ = 1.99, p = .047), and that it also interacted with disagreement valence and disagreement strength (β = -0.08 ± 0.03, $t(777)$ = -2.34, p = .018). As with the real social influence scores, this interaction effect was caused by a negative relation of the two variables exclusively found in strong positive disagreement trials (β = -0.04 ± 0.01, $t(259)$ = -2.78, p = .006). In all other types of disagreement trials, a positive association was found (moderate positive disagreement: β = 0.05 ± 0.02, $t(259)$ = 2.80, p = .006; strong negative disagreement: β = 0.02 ± 0.01, $t(259)$ = 1.67, p = .096; moderate negative disagreement: β = 0.03 ± 0.02, $t(259)$ = 1.64, p = .102). A complementary model in which strong positive disagreement trials were excluded confirmed the positive main of Germ Aversion on the social influence parameter δ (β = 0.04 ± 0.01, $t(259)$ = 3.36, p < .001). Bayesian analyses showed that this complementary model outperformed its baseline version by a factor of 5 (BF_{10} = 11.60 ± 2.01%). Similar results were obtained when the noise

parameter σ was taken as the dependent variable: the type 2 model including Germ Aversion as the indicator of perceived vulnerability to extrinsic morbidity risks was the only model that performed better than the baseline model. (*Germ Aversion* vs. *Baseline*: $BF_{10} = 1.72 \pm 6.87\%$). However, evidence in favor of an explanatory power of Germ Aversion on the noise parameter σ was weak (*Germ Aversion* vs. *Baseline*: $BF_{10} = 1.72 \pm 6.87\%$), even though the two variables were positively linked ($\beta = 0.12 \pm 0.05$, $t(259) = 2.56$, $p = .01$). Finally, the Perceived Infectability score had no effect neither on the social influence parameter δ, nor on the noise parameter σ (*BFs* < 1).

Laboratory study. When the social influence parameter δ was taken as the dependent variable, the type 1 model including Perceived Infectability as the indicator of perceived vulnerability to extrinsic morbidity risks was the only model that outperformed the baseline model (*Perceived Infectability* vs. *Baseline*: $BF_{10} = 1.28 \pm 5.66\%$). This model also outperformed type 1 models which included age or gender as predictor (*Perceived Infectability* vs. *age*: $BF_{10} = 3.27 \pm 5.37\%$; *Perceived Infectability* vs. *gender*: $BF_{10} = 1.70 \pm 6.61\%$) (Supplementary Figure S5.b). More specifically, the social influence parameter δ increased as long as participants felt more vulnerable to extrinsic morbidity risks ($\beta = 0.04 \pm 0.01$, $t(15) = 2.49$, $p = .025$). A weak improvement was also observed when the noise parameter σ was taken as the dependent variable (*Perceived Infectability* vs. *Baseline*: $BF_{10} = 1.20 \pm 5.23\%$). However, model parameters showed that the relation between the two variables was not significant ($\beta = 0.10 \pm 0.06$, $t(15) = 1.81$, $p = .09$). The Germ Aversion score had no effect neither on the social influence parameter δ, nor on the noise parameter σ (*BFs* < 1).

Analyses of computational data therefore suggest that, overall, the positive effect of indicators of perceived vulnerability to extrinsic morbidity risks on participants' susceptibility to social influence is mediated by an increased sensitivity to social feedbacks rather than by a corruption of their internal representations by noise.

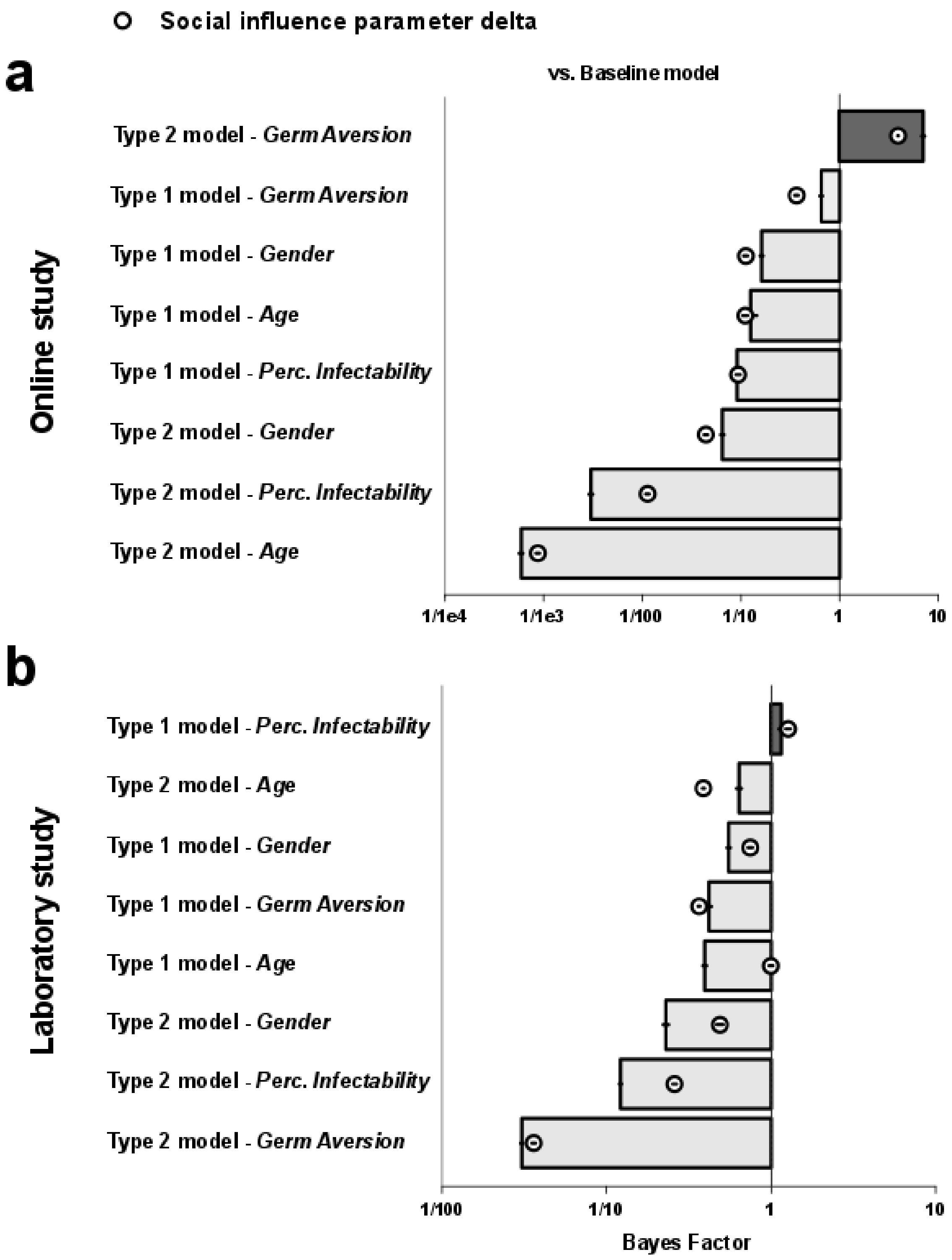

Supplementary Figure S5. Model selection analyses a. Online study. b. Laboratory study. Bayesian analyses of models with and without indicators of perceived vulnerability to extrinsic morbidity risks (Germ Aversion and Perceived Infectability), age or gender as predictor of social influence score (columns) and of the fitted social influence parameter delta (discs). The baseline model only includes disagreement valence and disagreement strength as within-subject factors; alternative models include indicators of perceived vulnerability to extrinsic morbidity risks, age or gender either as a main effect (type 1) or as a term interacting with disagreement valence and disagreement strength (type 2). A Bayes factor > 1 indicates greater evidence for the alternative model.

Multivariate decoding

Decoding public information as a function of disagreement valence and disagreement strength. Since analyses behavioural data collected in the laboratory showed that disagreement valence and disagreement strength had an independent effect on social influence scores, we studied the effect that these two factors may have on electroencephalographic activity separately using the same decoding methods as the one described in the main manuscript. The effect of disagreement valence was thus investigated by pooling together trials featuring a moderate negative disagreement with trials featuring a strong negative disagreement on one hand, and trials featuring a moderate positive disagreement with trials featuring a strong positive disagreement on the other hand. The effect of disagreement strength was investigated by pooling together trials featuring negative and positive disagreement of a moderate strength with trials featuring negative and positive disagreement of a bigger strength. Results fairly similar to those described in the main manuscript were obtained with decoders distinguishing – classifying – agreement trials from positive disagreement trials, negative disagreement trials, and strong disagreement trials (Supplementary Figure S6.a, S6.b). When moderate disagreement trials were entered in the classification pipeline however, the decoding sensitivity differed from chance during the time-period that was comprised between 200ms and 400ms post-stimulus. After that point, significance was only reached during a narrow period of 90ms starting 570ms post-stimulus.

Matrices of temporal generalization for positive, negative and strong disagreement trials all revealed two distinct processing stages. A first stage showed a sharp sensitivity peak around 300ms post-stimulus (*AUC peak* of .62 on average) which was caused by a negative deflection recorded within fronto-central sites of the scalp surface (Supplementary Figure S6.a, S6.b). The second processing stage was much more stationary, covered a wider time-window, and was caused by a negative differential activity in the vicinity of the right frontal electrodes and by a positive differential activity that was mainly distributed around the

occipito-parietal sites (Supplementary Figure S6.a, S6.b). This second processing stage was present for each type of disagreement trials. However, it was particularly marked for the decoding of strong disagreement trials, and almost absent for the decoding of moderate disagreement trials (Supplementary Figure S6.b).

In order to investigate which of the two processing stages better correlated with social influence scores, we performed correlation analyses similar to those described in the manuscript. First, the correlation analyses were applied on participants' social influence scores following negative disagreement trials and the corresponding decoding sensitivities computed at each time-point of the epoch. Results showed that, in the first processing stage, the two variables tended to correlate (cluster 1: $p^* = .07$; *mean* $r = .57$), and were found to be significantly related in the second stage of processing. A second cluster of correlations emerged in a time-window comprised between 535ms and 760ms post-stimulus ($p = .007$, *mean* $r = .55$). Of note is that while the fitted noise parameter σ never correlated with decoding sensitivities, patterns of correlations similar to those observed with real social influence scores were obtained with the fitted social influence parameter δ (1st stage cluster: $p^* = .058$, *mean* $r = .57$; 2nd stage cluster 1: $p^* = .03$, *mean* $r = .52$; 2nd stage cluster 2: $p^* = .02$, *mean* $r = .55$). No correlation between social influence scores obtained in positive disagreement trials and the corresponding decoding sensitivities was found.

Note finally that clusters of correlation found in moderate disagreement trials were not significant. In strong disagreement trials however, the correlation of social influence scores with decoding sensitivities was near-significance in the first ($p^* = .053$, *mean* $r = .51$) processing stage, and turned significant in the second processing stage ($p^* = .035$, *mean* $r = .47$).

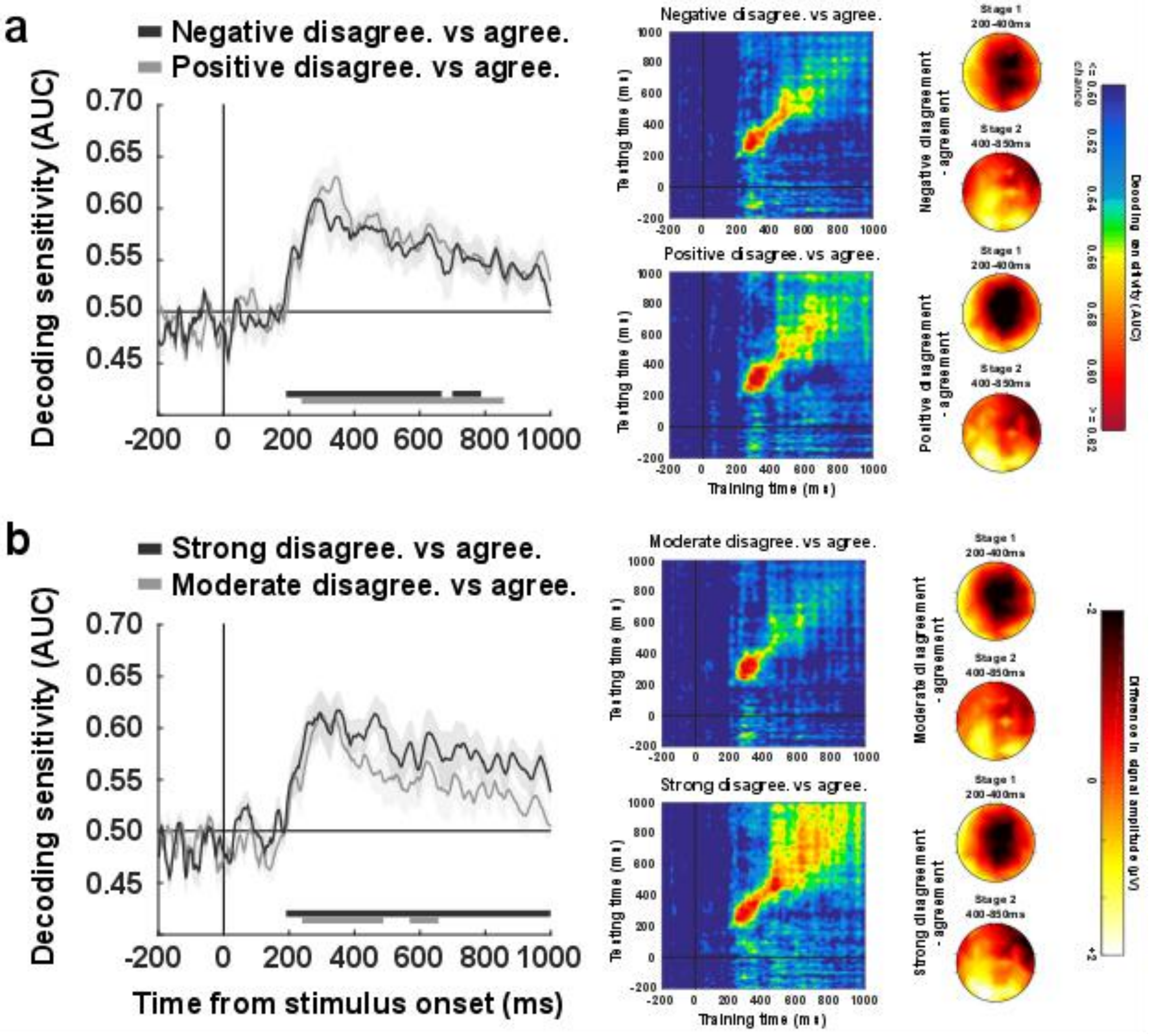

Supplementary Figure S6. Decoding stages of public information processing and temporal generalization. Sensitivity of the decoders that were trained to classify the various types of disagreement trials and agreement trials on the basis of the EEG activity recorded during the 1000ms that followed the exposure to public information. Decoders trained at each time point were tested on data from all other time points, revealing the presence of two distinct processing stages (stage 1 = 200-400ms post-stimulus; stage 2: 400-900ms post-stimulus). The diagonal (where testing time = training time) gives the curve for canonical decoders performance over time. Topographical maps of the differential EEG activity resulting from the contrast between the two classes of stimuli that were entered in each decoder are representative of processing stages 1 and 2. **a.** Disagreement trials are split as a function of their valence (positive, negative). **b.** Disagreement trials are split as a function of their strength (moderate, strong). Clusters of adjacent time-points in which the decoder's sensitivity significantly differed from chance are represented by the markers located up to the *x* axis.

Decoding public information as a function of perceived vulnerability to extrinsic morbidity risks. We also tested whether the participants' vulnerability to extrinsic morbidity risks was associated with the decoders' performance obtained at the two distinct stages of social feedback processing. For each stage (stage 1: from 200ms to 400ms post-stimulus; stage 2: from 400ms to 900ms post-stimulus), we ran correlation analyses between scores in Perceived Infectability or Germ Aversion and the decoders' sensitivities computed at each time-point of the epoch. We found that Perceived Infectability scores were positively related to decoding sensitivities obtained from the processing of strong disagreement trials, with a significant cluster of correlations emerging 755ms and ending 895ms post-stimulus ($p^* = .033$; mean $r = .52$). No other significant clusters of correlations were found.
